# Loss of FOXM1 in macrophages promotes pulmonary fibrosis by activating p38 MAPK signaling pathway

**DOI:** 10.1371/journal.pgen.1008692

**Published:** 2020-04-09

**Authors:** Chinmayee Goda, David Balli, Markaisa Black, David Milewski, Tien Le, Vladimir Ustiyan, Xiaomeng Ren, Vladimir V. Kalinichenko, Tanya V. Kalin

**Affiliations:** 1 Division of Pulmonary Biology, the Perinatal Institute of Cincinnati Children’s Hospital Research Foundation, Cincinnati, Ohio, United States of America; 2 Center for Lung Regenerative Medicine, the Perinatal Institute of Cincinnati Children’s Hospital Research Foundation, Cincinnati, Ohio, United States of America; HudsonAlpha Institute for Biotechnology, UNITED STATES

## Abstract

Idiopathic pulmonary fibrosis (IPF) is a chronic disease with high mortality and is refractory to treatment. Pulmonary macrophages can both promote and repress fibrosis, however molecular mechanisms regulating macrophage functions during fibrosis remain poorly understood. FOXM1 is a transcription factor and is not expressed in quiescent lungs. Herein, we show that FOXM1 is highly expressed in pulmonary macrophages within fibrotic lungs of IPF patients and mouse fibrotic lungs. Macrophage-specific deletion of *Foxm1* in mice (my*Foxm1*^-/-^) exacerbated pulmonary fibrosis. Inactivation of FOXM1 *in vivo* and *in vitro* increased p38 MAPK signaling in macrophages and decreased DUSP1, a negative regulator of p38 MAPK pathway. FOXM1 directly activated *Dusp1* promoter. Overexpression of DUSP1 in FOXM1-deficient macrophages prevented activation of p38 MAPK pathway. Adoptive transfer of wild-type monocytes to my*Foxm1*^-/-^ mice alleviated bleomycin-induced fibrosis. Altogether, contrary to known pro-fibrotic activities in lung epithelium and fibroblasts, FOXM1 has anti-fibrotic function in macrophages by regulating p38 MAPK.

## Author summary

Idiopathic pulmonary fibrosis (IPF) is a severe chronic lung disease, which is refractory to treatment. The environmental, age-related, and genetic factors increase susceptibility of the lung to injury. Repeated injury and deregulated repair result in scarring of the lung tissue and ultimate loss of respiratory function. Macrophages are involved at all stages of lung injury and repair, and can promote as well as repress fibrosis. However, molecular mechanisms regulating macrophage functions during fibrosis remain poorly understood. We have previously identified the FOXM1 protein to be highly increased in fibrotic lungs. High levels of FOXM1 in type II epithelial cells and fibroblasts exacerbated pulmonary fibrosis. In the present study, we found a novel and unexpected role of FOXM1. Contrary to pro-fibrotic functions of FOXM1 in epithelial cells and fibroblasts, FOXM1 has an anti-fibrotic role in macrophages. Mice lacking FOXM1 in macrophages developed severe pulmonary fibrosis following repeated lung injury with bleomycin. FOXM1 inhibited the p38 MAPK signaling pathway in pulmonary macrophages through transcriptional activation of DUSP1, a negative regulator of p38 MAPK pathway. Adoptive transfer of FOXM1-expressing monocytes protected FOXM1-deficient mice from bleomycin-induced pulmonary fibrosis. Altogether, FOXM1 has anti-fibrotic function in macrophages, limiting potential clinical use of FOXM1 inhibitors in IPF.

## Introduction

Idiopathic pulmonary fibrosis (IPF) is a chronic lung disease characterized by excessive accumulation of extracellular matrix, ultimately leading to disruption of gas exchange and respiratory functions. This disease remains poorly understood and is refractory to treatment. Inflammatory cells, including phagocytic cells like macrophages, have been shown to be intimately involved with all stages of lung injury and repair [1]. During acute wound healing, monocytes respond to a variety of chemo attractive cytokines and chemokines, are recruited to the lung and differentiate into macrophages [2]. Once present at the source of injury macrophages promote inflammation and wound healing to clear dead cells and to restore normal tissue homeostasis. Macrophage functions are diverse during fibrogenesis, consisting of both pro- and anti-fibrotic roles [1, 3, 4]. Macrophages produce anti-fibrotic mediators such as IL-13, ARG1 and FIZZ1 that repress inflammatory and fibrotic responses, and induce apoptosis of myofibroblasts [5–8]. Macrophages promote extracellular matrix (ECM) turnover by direct production of matrix metalloproteinases (MMPs), including MMP9 and MMP13 which degrade collagen [9, 10]. In contrast to anti-fibrotic functions, macrophages can promote fibrosis through secretion of pro-fibrotic and pro-inflammatory mediators including TGF-β1, PDGF and IL-1β [11–17]. Activation of p38 MAPK signaling pathway in pulmonary macrophages promotes lung fibrosis through secretion of IL-6, IL-1β, and TNF-α, all of which are the direct targets of p38 MAPK pathway [18]. While pulmonary macrophages can both promote and inhibit fibrogenesis, transcriptional mechanisms regulating these dual functions remain poorly understood.

The Forkhead box M1 (FOXM1) is a member of the Forkhead box (FOX) family of transcription factors. FOXM1 is highly expressed during embryogenesis but nearly absent from adult respiratory cell types, including pulmonary macrophages [19]. FOXM1 is aberrantly induced in the lungs of IPF patients and in the mouse fibrotic lungs after radiation- or bleomycin-induced lung injury [20]. Genetic deletion of *Foxm1* in alveolar epithelial type II cells (AECII) prevented both radiation- and bleomycin-induced lung fibrosis in mice, while over-expression of FOXM1 in AECII exacerbated fibrosis [20]. FOXM1 directly activated transcription of *Snail*, a critical regulator of epithelial to mesenchymal transition [20]. Furthermore, FOXM1 was found to be expressed in IPF fibroblasts [21, 22]. FOXM1 induced TGFβ-mediated differentiation of fibroblasts into myofibroblasts, and protected them from apoptosis. Deletion of *Foxm1* in myofibroblasts protected mice from bleomycin-induced pulmonary fibrosis and sensitized myofibroblasts to FasL-induced apoptosis [21]. In another study, FOXM1 increased expression of DNA repair proteins RAD51 and BRCA2 in IPF fibroblasts, and protected IPF fibroblasts from radiation-induced apoptosis [22]. All these published studies implicate FOXM1 in fibrotic responses and suggest that pharmacological inhibition of FOXM1 can be beneficial in IPF.

In the present study, we found a novel and unexpected role of FOXM1 in pulmonary fibrosis. Contrary to pro-fibrotic functions of FOXM1 in AECII and fibroblasts, FOXM1 has an anti-fibrotic role in macrophages. Mice lacking *Foxm1* in macrophages developed exacerbated pulmonary fibrosis following repeated intra-tracheal administration of bleomycin. FOXM1 inhibited the p38 MAPK signaling pathway in pulmonary macrophages through transcriptional activation of DUSP1, a negative regulator of p38 MAPK pathway [23]. Adoptive transfer of FOXM1-expressing monocytes protected FOXM1-deficient mice from bleomycin-induced pulmonary fibrosis. Altogether, FOXM1 has anti-fibrotic function in macrophages, limiting potential clinical use of FOXM1 inhibitors in IPF.

## Results

### FOXM1 expression is increased in pulmonary macrophages within human IPF and mouse fibrotic lungs

**Fig 1:**
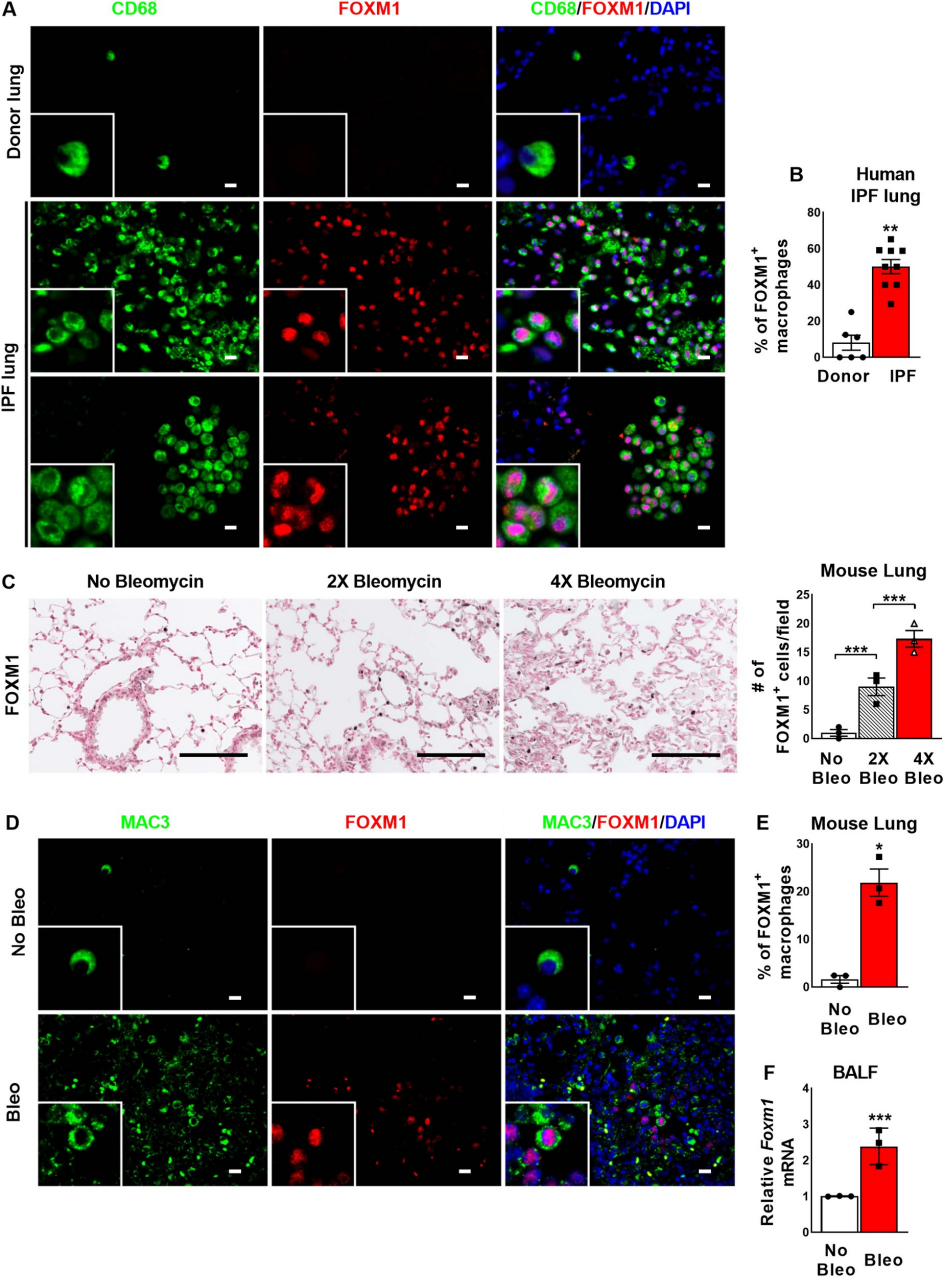
FOXM1 expression is induced in macrophages within human IPF and mouse fibrotic lungs. **(A)** FOXM1 (red) is not expressed in CD68^+^ macrophages (green) of control donor lungs (upper panels) but is upregulated in macrophages within fibrotic lesions (middle panels) and adjacent alveolar regions (bottom panels) of idiopathic pulmonary fibrosis (IPF) lungs. **(B)** Percent of FOXM1^+^/CD68^+^ double positive cells were counted in 5 random fields and presented as mean ± SEM. Scale bar = 20*μ*m. **(C)** FOXM1 (brown) is not expressed in control mouse lungs and gradually increased during the progression of bleomycin-induced pulmonary fibrosis in mice. Numbers of FOXM1^+^ cells were counted in 5 random fields and presented as mean ± SEM. N = 3 mice per group. Scale bar = 100*μ*m. **(D)** FOXM1 (red) is not expressed in MAC3^+^ macrophages (green) of control mouse lungs (upper panels) but is upregulated in macrophages within fibrotic lesions and adjacent alveolar regions of bleomycin-injured fibrotic lungs (bottom panels). **(E)** Percent of FOXM1^+^/MAC3^+^ double positive cells were counted in 5 random fields and presented as mean ± SEM. N = 3 mice per group. Scale bar = 20*μ*m. **(F)** *Foxm1* mRNA is increased in BALF cells from bleomycin-treated mice as determined by qRT-PCR. *Actb* mRNA was used for normalization. N = 3 mice per group. * = *P <* 0.05. *** = P < 0.0001.

The macrophage-specific expression of FOXM1 in the lung tissue of IPF patients was visualized by a co-staining of lung sections for FOXM1 and the macrophage marker CD68. Fibrotic lesions in IPF lungs were identified by H&E staining and immunostaining for αSMA (S1 Fig). In human donor lungs without IPF, less than 10% of macrophages were positive for FOXM1 (Fig 1A, upper panels). In contrast, lungs of IPF patients showed abundant expression of FOXM1 in macrophages within fibrotic lesions as well as adjacent alveolar regions (Fig 1A, middle and bottom panels). Approximately 55% of the CD68^+^ cells were positive for FOXM1 in IPF lungs (Fig 1B). Thus, FOXM1 expression is increased in macrophages within human IPF lungs.

Expression of FOXM1 in macrophages was examined in mouse lungs during progression of bleomycin-induced pulmonary fibrosis. Wild type mice were treated with bleomycin via intra-tracheal instillation once every week for four consecutive weeks and mice were sacrificed one week after the final bleomycin treatment. Saline-treated mice were used as controls. Immunostaining for FOXM1 showed the presence of only single FOXM1-positive cells in control mouse lungs, and a gradual increase in the number of FOXM1-positive cells after bleomycin treatments (Fig 1C). Expression of FOXM1 in macrophages was visualized by co-staining of lung sections for FOXM1 and MAC3. Similar to human donor lungs, less than 10% of macrophages within normal mouse lungs were positive for FOXM1 (Fig 1D and 1E). The percentage of macrophages expressing FOXM1 was significantly increased in bleomycin-treated mouse lungs (Fig 1D and 1E), a finding consistent with the results from the human IPF studies (Fig 1B). In addition to increased FOXM1 staining, *Foxm1* mRNA was increased in macrophages isolated from bronco-alveolar lavage fluid (BALF) of bleomycin-treated mice (Fig 1F). *Foxm1* mRNA was also increased in neutrophils purified from bleomycin-treated lungs (S2 Fig). Altogether, FOXM1 mRNA and protein are increased in pulmonary macrophages within mouse and human fibrotic lungs.

### Genetic deletion of *Foxm1* in myeloid cells exacerbates bleomycin-induced pulmonary fibrosis

**Fig 2:**
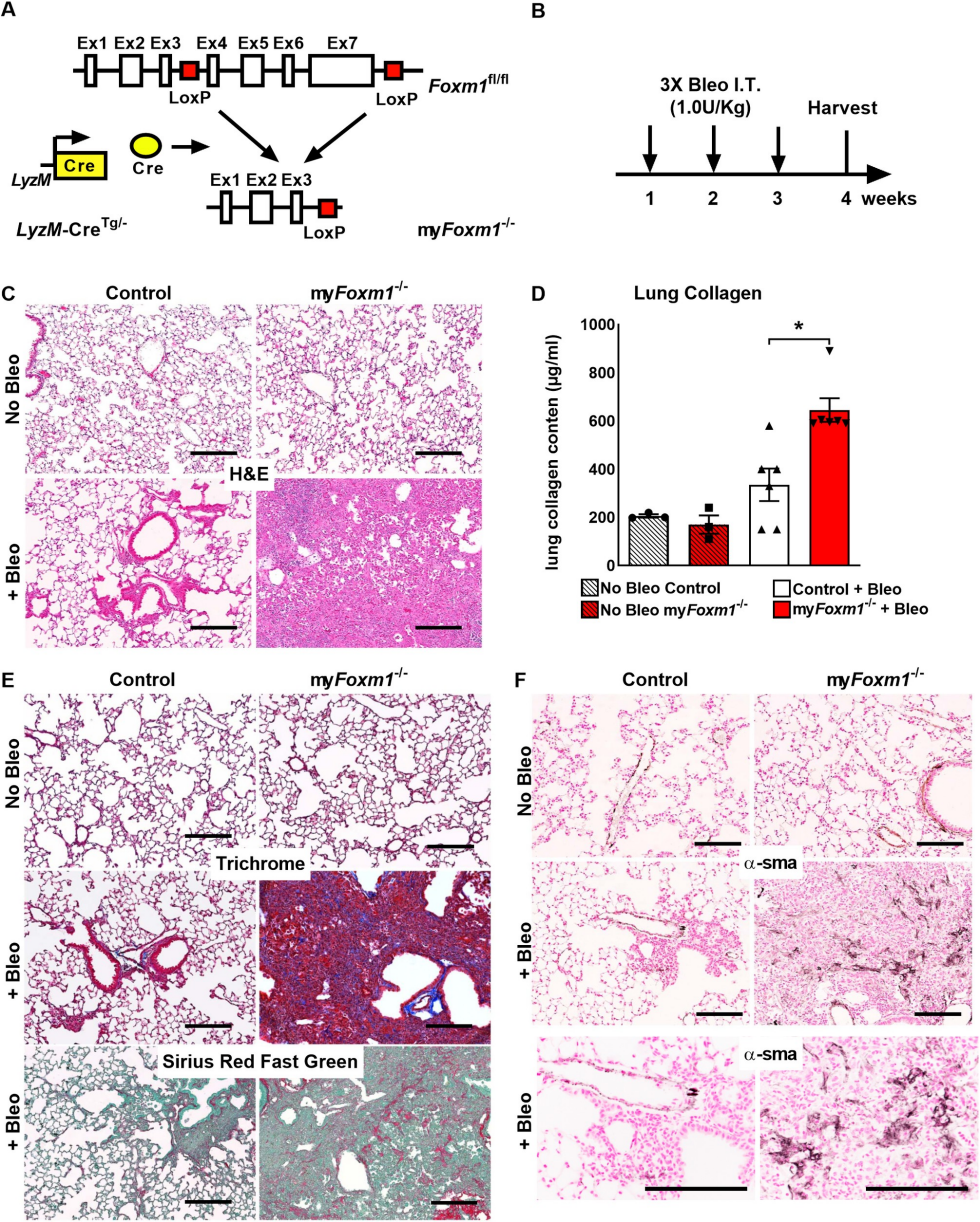
Conditional deletion of *Foxm1* in myeloid cells exacerbates bleomycin-induced pulmonary fibrosis in mice. **(A)** Schematic diagram shows breeding strategy for the deletion of *Foxm1* from myeloid cells (my*Foxm1*^-/-^). **(B)** Schematic representation for bleomycin-induced pulmonary fibrosis in mice. 8–10 weeks old my*Foxm1*^-/-^ and control mice were treated with bleomycin intratracheally (1U/kg; once a week for three weeks). Mice were sacrificed, and lungs were collected one week after the final bleomycin treatment. **(C)** Deletion of *Foxm1* from myeloid cells does not affect lung architecture in uninjured mice as shown by H&E staining (top panels). Increased pulmonary fibrosis was detected in bleomycin-treated my*Foxm1*^-/-^ mice compared to control bleomycin-treated mice (bottom panels). Scale bar = 200*μ*m. **(D)** Increased lung collagen content, as quantified by Sircol assay, was detected in bleomycin-treated my*Foxm1*^-/-^ mice compared to control (n = 6 mice per group). **(E)** Increased lung collagen deposition in bleomycin-treated my*Foxm1*^-/-^ mice was shown using Masson’s trichrome and Sirius red fast green staining. Scale bar = 200*μ*m. **(F)** Deletion of *Foxm1* from myeloid cells increased number of αSMA^+^ myofibroblasts in bleomycin-treated my*Foxm1*^-/-^ mice. No changes in the number of αSMA^+^ lung myofibroblasts were found in uninjured my*Foxm1*^-/-^ mice compared to control lungs (n = 5 mice per group). Scale bar = 200*μ*m. * = *P <* 0.05.

To determine FOXM1 requirements in macrophages during progression of pulmonary fibrosis, we utilized a previously generated myeloid-specific *Foxm1* knockout mouse model (*LysM-Cre*^tg/-^*;Foxm1*^fl/fl^; abbreviated as my*Foxm1*^-/-^) [24, 25]. Following Cre-mediated recombination of the *Foxm1-flox* allele, exons 4–7 encoding the DNA binding and transcriptional activation domains of FOXM1 protein were deleted in myeloid cells, including monocytes, macrophages and neutrophils (Fig 2A) [25]. The my*Foxm1*^-/-^ and control *Foxm1*^fl/fl^ mice were treated with bleomycin via intra-tracheal instillation once every week for three consecutive weeks (Fig 2B). The lungs of uninjured control and my*Foxm1*^-/-^ mice had normal lung architecture (Fig 2C, top panels), indicating that FOXM1 expression in myeloid cell lineage is dispensable for lung development. Following chronic bleomycin injury, deletion of *Foxm1* increased the severity of pulmonary fibrosis as shown by H&E staining (Fig 2C and S3 Fig) and Sircol assay measuring collagen content (Fig 2D). Masson’s trichrome and Sirius red/ Fast green staining showed increased collagen depositions in bleomycin-treated my*Foxm1*^-/-^ lungs compared to controls (Fig 2E). We next assessed the presence of myofibroblasts using immunostaining for α-Smooth muscle actin (αSMA). Without bleomycin injury, no difference in αSMA staining was observed in control and my*Foxm1*^-/-^ lungs (Fig 1F, upper panels). Bleomycin treatment increased the number of αSMA^+^ myofibroblasts in the lungs of my*Foxm1*^-/-^ mice compared to bleomycin-treated controls (Fig 2F, middle, and bottom panels), a finding consistent with elevated collagen levels in the lung tissue (Fig 2D and 2E). Thus, deletion of *Foxm1* from myeloid cells increased lung fibrosis in bleomycin-treated mice.

### Deletion of *Foxm1* increases expression of pro-inflammatory cytokines in pulmonary macrophages

**Fig 3:**
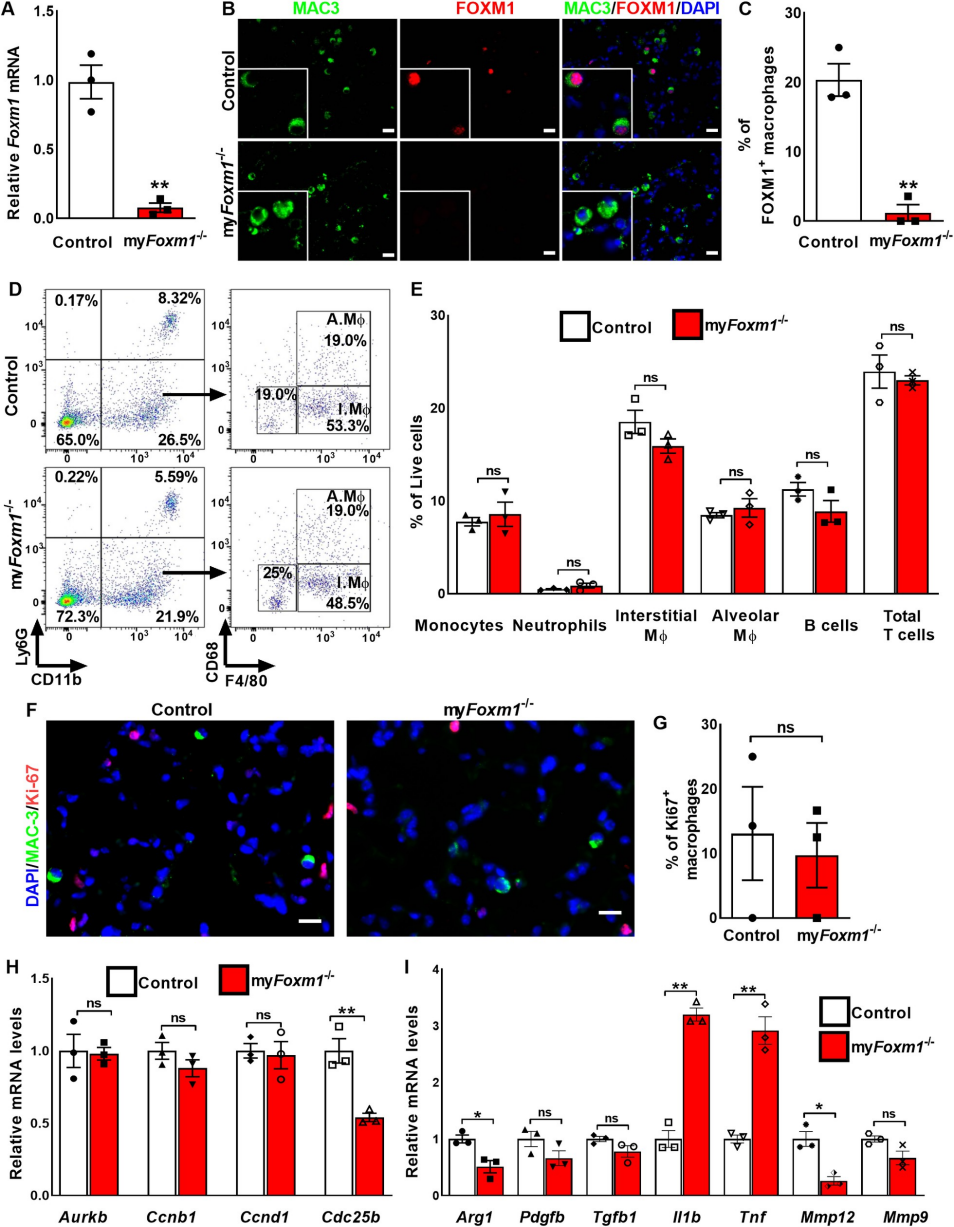
Deletion of *Foxm1* from macrophages increases expression of pro-fibrotic cytokines without affecting number of myeloid cells. **(A)** *Foxm1* mRNA levels were decreased in BALF cells isolated from bleomycin-treated my*Foxm1*^-/-^ lungs as determined by qRT-PCR. *Actb* mRNA was used for normalization (n = 3). ** = P < 0.01. **(B)** Co-localization studies showed efficient deletion of FOXM1 (red) from MAC-3^+^ macrophages (green). **(C)** Percent of FOXM1^+^/MAC3^+^ double positive cells were counted in 5 random fields and presented as mean ± SEM. N = 3 mice per group. Scale bar = 20*μ*m. **(D)** Deletion of FOXM1 from myeloid cells did not affect the number of immune cells in bleomycin-treated lungs as demonstrated using flow cytometry analysis. **(E)** Percentages of Monocytes (CD45^+^Ly6G^-^CD11b^+^F4/80^low^), Neutrophils (CD45^+^ CD11b^+^ Ly6G^+^), Interstitial macrophages (CD45^+^Ly6G^-^CD11b^+^F4/80^high^CD68^low^), Alveolar macrophages (CD45^+^Ly6G^-^CD11b^+^F4/80^high^CD68^high^), B cells (CD45^+^B220^+^), and T cells (CD45^+^CD3^+^) were not changed in bleomycin-treated control and my*Foxm1*^-/-^ mice. N = 3 mice per group. **(F-G)** Co-localization studies showed no difference in the number of Ki-67^+^ (red) MAC-3^+^ macrophages (green) in bleomycin-treated my*Foxm1*^-/-^ mice. N = 3 mice per group. Scale bar = 20*μ*m. **(H)** *Aurkb*, *Ccnb1*, and *Ccnd1* mRNAs were unchanged, while *Cdc25b* mRNA was decreased in BALF cells from bleomycin-treated my*Foxm1*^-/-^ mice. *Actb* mRNA was used for normalization. N = 3 mice per group. **(I)** mRNAs of anti-fibrotic *Arg1* and *Mmp12* were decreased, while mRNAs of pro-fibrotic *Il1b*, and *Tnf* were increased in BALF cells from bleomycin-treated my*Foxm1*^-/-^ mice as determined by qRT-PCR. *Actb* mRNA was used for normalization. N = 3 mice per group. All data are mean ± SEM. *P <0.05, **P <0.01, by Student’s t-test.

To examine the efficiency of *Foxm1* deletion, BALF cells containing >90% of macrophages were isolated from my*Foxm1*^-/-^ and control mice and used for qRT-PCR. *Foxm1* mRNA was decreased by 94% in BALF cells from my*Foxm1*^-/-^ mice, confirming efficient deletion of *Foxm1* by the *LyzM-cre* transgene (Fig 3A). We also assessed FOXM1 protein in pulmonary macrophages by co-staining lung sections for FOXM1 and MAC-3. After bleomycin treatment, FOXM1 protein was often found in the nuclei of macrophages in control *Foxm1*^fl/fl^ lungs (Fig 3B, upper panels). In contrast, in bleomycin-treated my*Foxm1*^-/-^ lungs, macrophages were negative for FOXM1 (Fig 3B, bottom panels). The percentage of FOXM1-positive macrophages was significantly decreased in bleomycin-treated my*Foxm1*^-/-^ lungs compared to controls (Fig 3C). Thus, FOXM1 was efficiently deleted in macrophages in my*Foxm1*^-/-^ lungs.

Since FOXM1 increased the recruitment of macrophages during lung carcinogenesis [24] and liver injury [26], we used flow cytometry to examine inflammatory response in bleomycin-treated mouse lungs (Fig 3D and S4A Fig). Surprisingly, deletion of *Foxm1* did not change the percentages of monocytes, interstitial and alveolar macrophages in the bleomycin-injured lung tissue (Fig 3E). mRNAs of *Cx3cr1* and *Ccr2*, the chemokine receptors critical for recruitment of macrophages to the lung tissue [24, 27] were not changed in bleomycin-treated my*Foxm1*^-/-^ lungs (S4B Fig). The percentages of T and B lymphocytes were also similar in bleomycin-treated control and my*Foxm1*^-/-^ lungs (Fig 3E and S4C Fig). Numbers of neutrophils were low in bleomycin-treated lungs (Fig 3E). Thus, deletion of *Foxm1* from myeloid cells did not change the percentages of inflammatory cells in fibrotic lungs. Since FOXM1 induces cellular proliferation in various cell types [19], co-localization of Ki-67 and MAC3 was performed to assess the number of proliferating lung macrophages. No difference in the number of Ki-67^+^/Mac-3^+^ macrophages was found in bleomycin-treated my*Foxm1*^-/-^ lungs compared to bleomycin-treated control lungs (Fig 3F and 3G). mRNAs of proliferation-specific *Aurkb*, *Ccnb1*, and *Ccnd1* were unchanged, whereas *Cdc25b* mRNA was decreased in BALF macrophages from bleomycin-treated my*Foxm1*^-/-^ mice as determined by qRT-PCR (Fig 3H).

Interestingly, mRNAs of anti-fibrotic *Arg1* and *Mmp12* were decreased while mRNAs of pro-fibrotic *Il1b* and *Tnf* were increased in BALF macrophages from bleomycin-treated my*Foxm1*^-/-^ mice (Fig 3I). Since ARG1-positive macrophages were shown to protect liver from fibrosis [8], we examined ARG1 expression in fibrotic mouse lungs. Deletion of *Foxm1* decreased the number of ARG1^+^ cells (S5A Fig). *Agr1* mRNA was decreased in macrophages isolated from BALF and from lung tissue using FACS sorting (S5B Fig and S5C Fig). Interestingly, *Mrc1* mRNA, encoding a type I membrane receptor that mediates the endocytosis of glycoproteins by macrophages [28], was decreased in lung interstitial and BALF macrophages isolated from bleomycin-treated my*Foxm1*^-/-^ mice (S6A Fig and S6B Fig). While *Tgfb1* mRNA was increased in the whole lung RNA from bleomycin-treated my*Foxm1*^-/-^ mice (S6C Fig), *Tgfb1* was unchanged in FOXM1-deficient macrophages (Fig 3I and S6D Fig). To compare the functional ability of macrophages to degrade collagen, we generated bone-marrow derived macrophages from control and my*Foxm1*^-/-^ mice. Consistent with decreased *Mrc1* expression, the collagen degradation ability of FOXM1-deficient macrophages was significantly decreased compared to WT macrophages as shown by collagen degradation assay (S6E Fig, S6F Fig and S6G Fig).

Thus, genetic deletion of *Foxm1* from myeloid cells did not change the percentage of inflammatory cells in the bleomycin-treated lungs, but altered expression of fibrosis-associated genes, and decreased the ability of macrophages to degrade collagens.

### Cre-mediated deletion of *Foxm1* from macrophages activates p38 MAPK pathway

**Fig 4:**
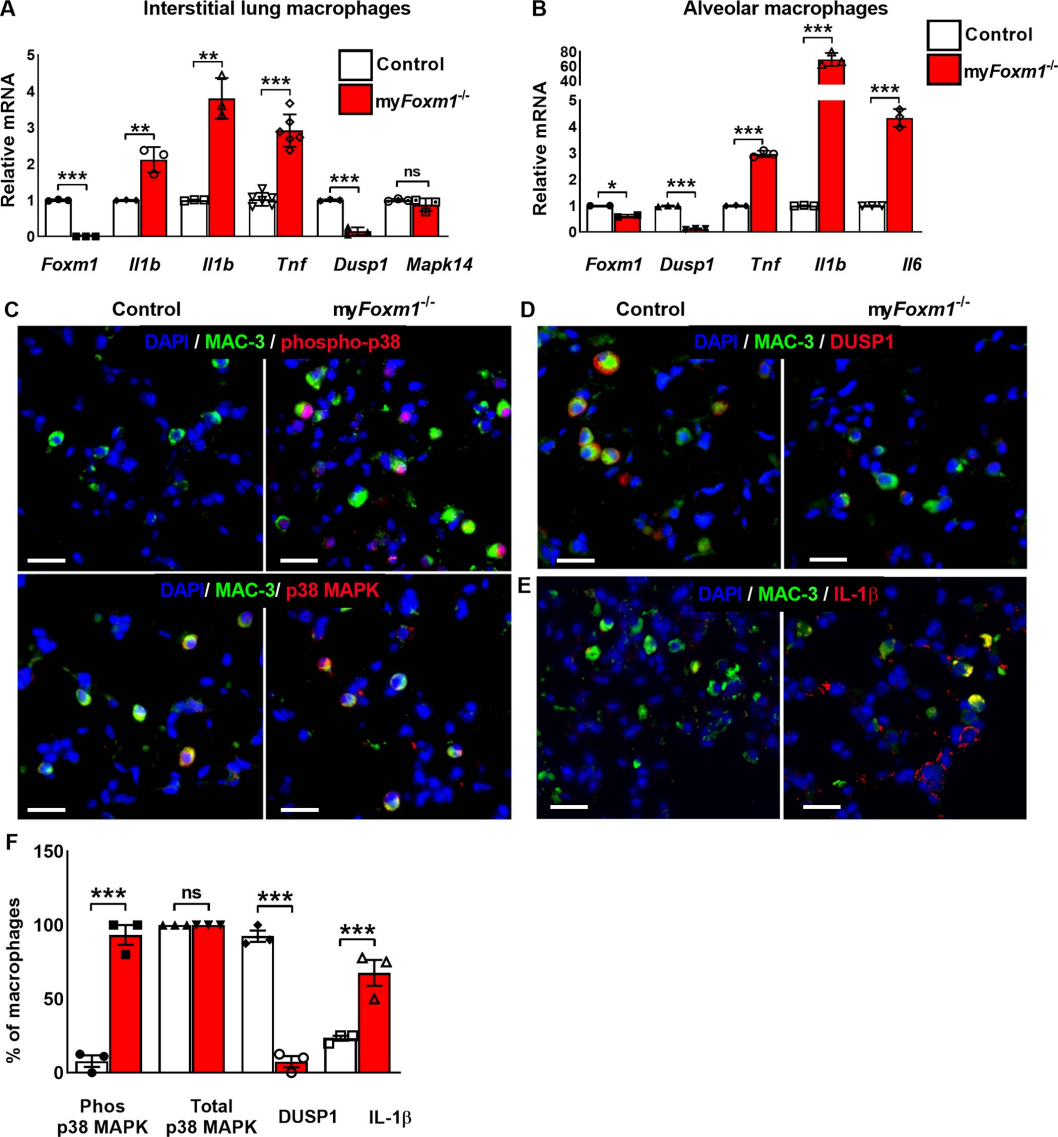
Deletion of *Foxm1* from macrophages induces activation of p38 MAPK pathway. **(A-B)** Interstitial and alveolar macrophages were isolated from bleomycin-treated my*Foxm1*^-/-^ and control lungs using FACS sorting. qRT-PCR shows decreased *Foxm1*, and *Dusp1* mRNAs and increased *Tnf*, *Ccl5*, *Il1β*, and *Il6* mRNAs in flow-sorted interstitial and alveolar macrophages from bleomycin-treated my*Foxm1*^-/-^ mice. *Actb* mRNA was used for normalization. N = 3 mice per group. **(C)** Increased expression of phosphorylated p38-MAPK (red) in MAC-3^+^ (green) macrophages was shown in bleomycin-treated my*Foxm1*^-/-^ mice compared to control (upper panels). No difference in total p38-MAPK (red) was observed in macrophages in bleomycin-treated my*Foxm1*^-/-^ mice compared to control (lower panels). **(D)** Co-localization studies showed decreased expression of DUSP1 (red) in MAC-3^+^ (green) macrophages in bleomycin-treated my*Foxm1*^-/-^ mice. **(E)** Co-localization studies show increased expression of IL-1β (red) in MAC-3^+^ (green) macrophages in bleomycin-treated my*Foxm1*^-/-^ mice. Scale bar = 50*μ*m. **(F)** Percent of phospho-p38^+^/MAC3^+^, Total p38^+^/MAC3^+^, DUSP1^+^/MAC3^+^ and IL-1β^+^/MAC3^+^ double positive cells were counted in 5 random fields and presented as mean ± SEM. N = 3 mice per group. *P <0.05, **P <0.01, ***P <0.001 by Student’s t-test.

Next, we examined gene expression in different subsets of lung macrophages. Interstitial (CD45^+^CD11b^+^Ly6G^-^F4/80^hi^CD68^low^) and alveolar (CD45^+^CD11b^+^Ly6G^-^F4/80^hi^CD68^hi^) macrophages were isolated from bleomycin-treated control and my*Foxm1*^-/-^ lungs using FACS sorting (Fig 4A and 4B). While the efficiency of *Foxm1* deletion was better in interstitial macrophages, *Foxm1* mRNA was significantly decreased in both macrophage subsets (Fig 4A and 4B). Consistent with the BALF data (Fig 3I), mRNAs of *Il1b*, *Il6* and *Tnf* were increased in both interstitial and alveolar macrophages isolated from my*Foxm1*^-/-^ lungs (Fig 4A and 4B). Since these cytokines are known downstream targets of p38 MAPK pathway [29], we examined phosphorylation of p38 MAPK (Thr180/Tyr182), which activates p38 MAPK protein leading to increased expression of pro-inflammatory cytokines [30]. Phosphorylation of p38 (phospho-p38) was increased in pulmonary macrophages in bleomycin-treated my*Foxm1*^-/-^ mice compared to controls (Fig 4C top panels and Fig 4F). Total p38 MAPK was similar in control and my*Foxm1*^-/-^ macrophages (Fig 4C bottom panels and Fig 4F). We next analyzed the expression of DUSP1, a dual specificity phosphatase that dephosphorylates p-p38 MAPK and acts as a negative regulator of the p38 MAPK signaling pathway [23]. *Dusp1* mRNA was decreased in both interstitial and alveolar macrophages isolated from bleomycin-treated my*Foxm1*^-/-^ mice (Fig 4A and 4B). Immunostaining for DUSP1 and the number of DUSP1-positive macrophages were also decreased in my*Foxm1*^-/-^ lungs compared to controls (Fig 4D and 4F). In addition, we found the increased number of IL-1β-positive macrophages in bleomycin-treated my*Foxm1*^-/-^ lungs compared to control lungs (Fig 4E and 4F). Thus, deletion of *Foxm1* increased phosphorylation of p38 MAPK, decreased DUSP1 and increased expression of pro-inflammatory cytokines that are downstream targets of the p38 MAPK signaling pathway.

### Inhibition of FOXM1 *in vitro* activates p38 MAPK pathway

**Fig 5:**
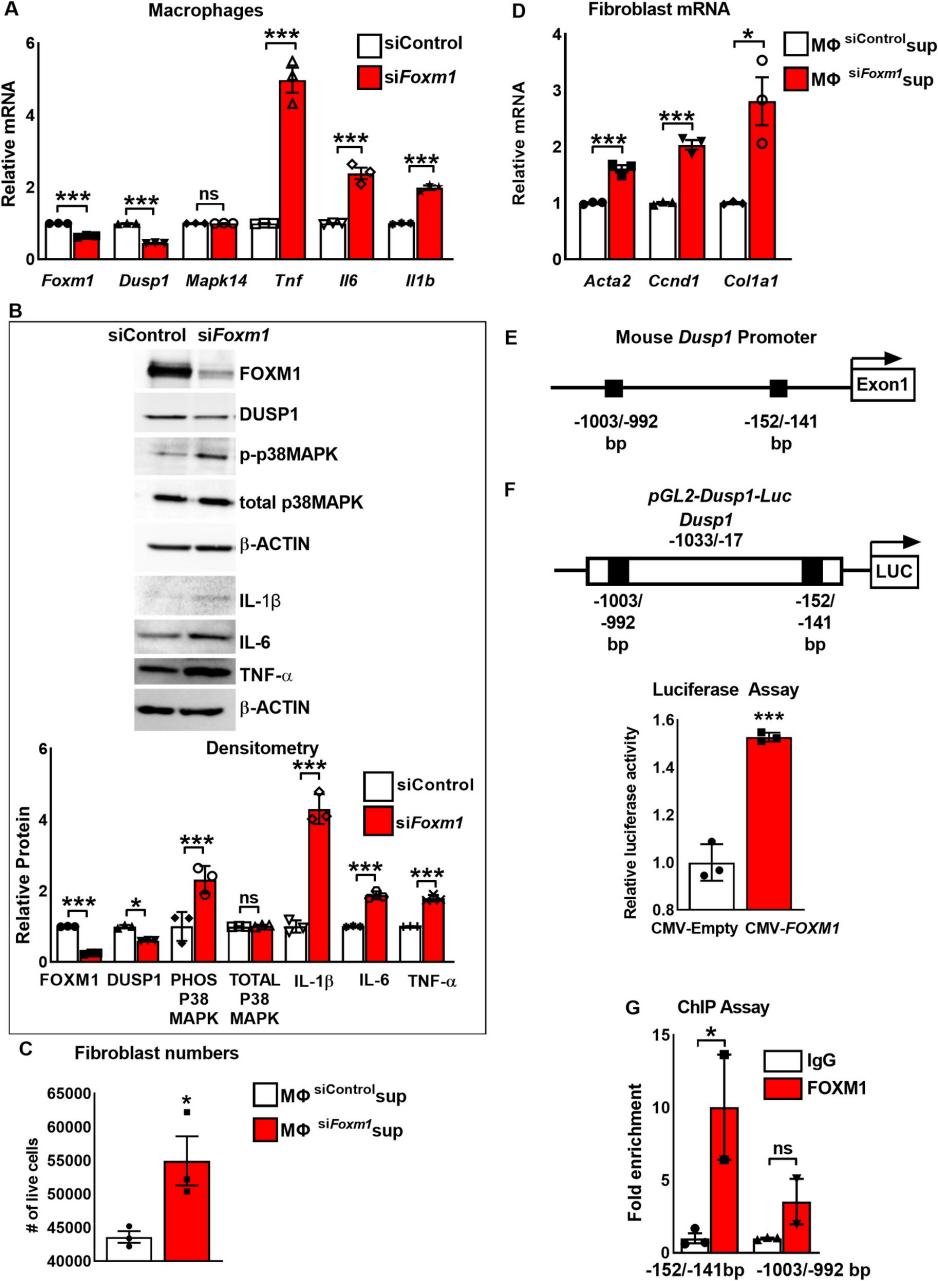
FOXM1 directly binds to and induces transcriptional activity of *Dusp1* promoter in macrophages. **(A)** Depletion of *Foxm1* in macrophages decreased *Dusp1* mRNA and increased *Tnf*, *Il6* and *Il1b* mRNA. *Mapk14* mRNA was unchanged. RAW264.7 macrophages were transfected with non-targeting siRNA (siControl) or si*Foxm1*. mRNA levels were analyzed by qRT-PCR. *Actb* mRNA was used for normalization (n = 3). **(B)** Depletion of *Foxm1* from macrophages results in activation of p38 MAPK signaling pathway. RAW264.7 macrophages were transfected with non-targeting siRNA (siControl) or si*Foxm1* and protein levels were analyzed by Western blot (top panel). FOXM1, DUSP1 protein levels were decreased and phospho-p38 MAPK, TNF-α, IL-1β and IL-6 levels were increased in *Foxm1*-deficient RAW264.7 macrophages. No changes were detected in total p38 MAPK. β-ACTIN was used as loading control. Blots are representative of three independent experiments. Densitometry was used to quantify Western blot results (bottom panel). Protein levels were normalized to β-ACTIN. Protein expression is presented as fold change over siControl transfected macrophages. **(C)** Supernatant from *Foxm1*-depleted RAW264.7 macrophages increases the number of viable 3T3 fibroblasts. 3T3 cells were cultured in supernatant obtained from RAW264.7 cells transfected with control siRNA or si*Foxm1*. 48 hours after transfection, the number of live cells was determined by trypan blue (n = 3). **(D)** Supernatant from *Foxm1*-depleted RAW264.7 macrophages increases *Acta2*, *Ccnd1*, and *Col1a1* mRNA levels in 3T3 fibroblasts. mRNA levels were measured by qRT-PCR. *Actb* mRNA was used for normalization (n = 3). **(E)** Schematic representation of the mouse *Dusp1* promoter region with potential FOXM1 DNA-binding sites (black boxes). **(F)** Schematic representation of the *pGL2-Dusp1-Luc* construct containing the *Dusp1* promoter region. The mouse *Dusp1* promoter (-1033/-17 bp) was cloned into the *pGL2-*LUC vector and co-transfected with CMV-Empty vector (CMV-EV) or CMV-*FOXM1*. Luciferase assay shows that FOXM1 transcriptionally activates the *Dusp1* promoter. Data represent mean ± SEM of three independent experiments. **(G)** ChIP assays shows direct binding of FOXM1 to the -152/-141bp *Dusp1* promoter region. FOXM1 binding is shown relative to immunoglobulin G (IgG). Data represent mean ± SEM of two independent experiments. * = *P >* 0.05; ** = *P* > 0.01, *** = *P* < 0.001, by Student’s t-test.

The effect of *Foxm1* deletion on p38 MAPK pathway was tested *in vitro* using RAW 264.7 macrophage cell line. Expression of *Foxm1* in RAW 264.7 cells was inhibited using siRNA-mediated gene silencing (si*Foxm1*), which reduced *Foxm1* mRNA (Fig 5A) and FOXM1 protein (Fig 5B). Consistent with our *in vivo* studies, knockdown of *Foxm1* reduced *Dusp1* and increased *Tnf*, *Il6 and Il1b* mRNAs (Fig 5A). Furthermore, knockdown of *Foxm1* increased protein levels of phosphorylated p38 MAPK without affecting total p38 MAPK protein as shown by Western blots (Fig 5B). The protein levels of TNF-α, IL-6, IL-1β were also increased after inhibition of FOXM1, consistent with the increased phospho-p38 MAPK (Fig 5B). Interestingly, supernatant from FOXM1-deficient macrophages increased the number of viable fibroblasts after 48 days in culture (Fig 5C) and increased fibroblast activation as shown by increased expression of *Acta2*, *Col1a1* and *Ccnd1* (Fig 5D). Neutralizing antibodies against IL-1β or IL-6 decreased number of fibroblasts and their activation after treatment with supernatant derived from FOXM1-deficient macrophages (S7A Fig, S7B Fig and S7C Fig). Neutralizing antibodies against TNF-α decreased the number of fibroblasts (S7A Fig) but did not change activation of fibroblasts (S7B Fig and S7C Fig). Thus, FOXM1-deficient macrophages directly increase fibroblasts numbers and activation, at least in part, through IL-1β, IL-6 and TNF-α. Altogether, inhibition of FOXM1 *in vitro* and *in vivo* reduced DUSP1 expression, increased p38 MAPK activation and increased expression of pro-fibrotic cytokines.

### FOXM1 directly binds to and induces transcriptional activity of *Dusp1* promoter

Since inhibition of FOXM1 decreased DUSP1 protein and mRNA *in vitro* and *in vivo*, we investigated whether FOXM1 transcriptionally activates *Dusp1* promoter. Two potential FOXM1 binding sites were identified in the -1.0-kb promoter region of the mouse *Dusp1* gene (Fig 5E). To test whether FOXM1 directly activates *Dusp1* transcription, we cloned the *-*1033/-17 bp *Dusp1* promoter region, which contains the -1003/-992 bp and -152/-141 bp FOXM1 binding sites, into the *pGL2-basic* luciferase (LUC) vector to generate *pGL2-Dusp1–Luc* reporter (Fig 5F). This reporter was co-transfected with CMV-Empty or CMV-*FOXM1* expression plasmids and transcriptional activation of the reporter was tested by measuring LUC activity. CMV-*FOXM1* plasmid induced transcriptional activity of the *Dusp1* promoter region (Fig 5F). Furthermore, chromatin immunoprecipitation (ChIP) was performed using RAW264.7 macrophages to identify functional FOXM1 binding sites in the *Dusp1* promoter. FOXM1 specifically bound to the -152/-141 bp, but not to -1003/-992 bp region of the *Dusp1* promoter (Fig 5G). These data indicate that FOXM1 directly binds to and transcriptionally activates *Dusp1* promoter in macrophages.

### Overexpression of DUSP1 decreases activation of p38 MAPK pathway in FOXM1-deficient macrophages

**Fig 6:**
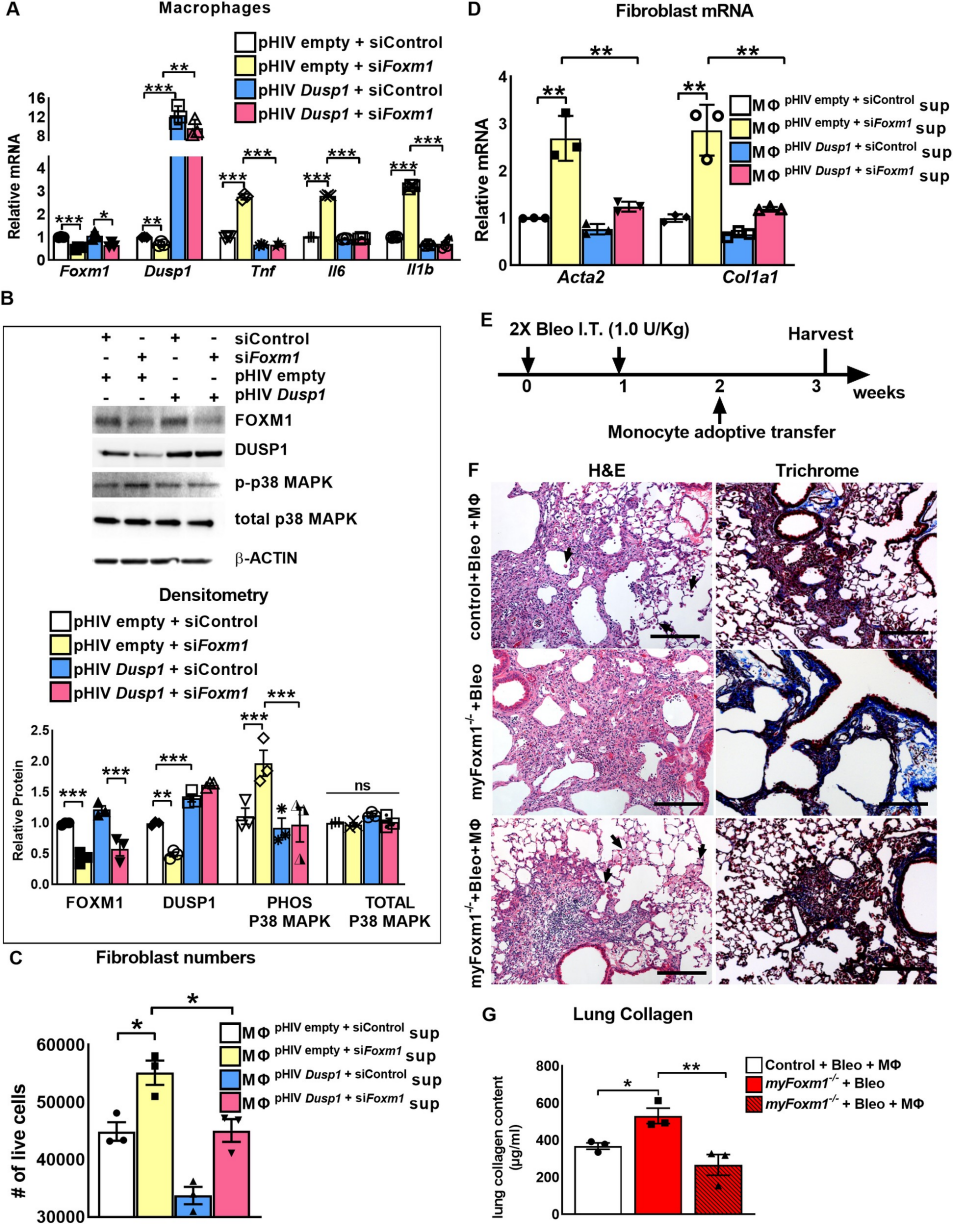
Adoptive transfer of wild-type macrophages reduces lung fibrosis in myFoxm1^-/-^ mice. **(A)** Overexpression of *Dusp1* decreases expression of *Tnf*, *Il6* and *Il1b* in FOXM1-deficient macrophages. RAW264.7 macrophages were co-transfected with non-targeting siRNA (siControl) or si*Foxm1*, and pHIV-empty vector or pHIV-*Dusp1*. mRNAs were analyzed by qRT-PCR. Overexpression of *Dusp1* decreased *Tnf*, *Il6* and *Il1b* mRNAs in si*Foxm1* transfected macrophages as shown by qRT-PCR. *Actb* mRNA was used for normalization (n = 3). **(B)** Overexpression of *Dusp1* in FOXM1-deficient macrophages decreases activation of p38 MAPK pathway. RAW264.7 macrophages were co-transfected with non-targeting siRNA (siControl) or si*Foxm1*, and pHIV-empty vector or pHIV-*Dusp1* and protein levels were analyzed by Western blot. Expression of DUSP1 in *Foxm1*-depleted RAW264.7 macrophages decreased phospho-p38 MAPK protein levels. No changes were detected in total p38 MAPK protein levels. β-ACTIN was used as loading control. Graph shows densitometry quantification of Western blots. Protein levels were normalized to β-ACTIN. Protein expression is presented as fold change over pHIV empty + siControl transfected cells. Blots are representative of three independent experiments. **(C-D)** Supernatant from *Dusp1* overexpressing *Foxm1*-deficient macrophages decreases fibroblast number and activation. 3T3 fibroblasts were cultured in supernatant obtained from RAW264.7 macrophages co-transfected with control siRNA or si*Foxm1*, and pHIV-empty vector or pHIV-*Dusp1*. 48 hours after transfection number of live cells were counted by trypan blue (n = 3). Expression of *Acta2*, *Ccnd1*, and *Col1a1* mRNAs in fibroblasts was measured by qRT-PCR. *Actb* mRNA was used for normalization (n = 3). **(E)** Experimental design of adoptive transfer. **(F)** Representative H&E images (left) and trichrome images (right) show significant reduction in fibrosis in my*Foxm1*^-/-^ mice upon adoptive transfer of FOXM1-expressing WT monocytes (n = 3 mice per group). Scale bar = 200*μ*m. **(G)** Lung collagen content was measured using the Sircol assay and revealed that transfer of FOXM1-expressing WT monocytes into my*Foxm1*^-/-^ mice significantly reduced bleomycin-induced lung fibrosis. * = *P <* 0.05, ** = *P* <0.01, *** = *P* < 0.001, by Student’s t-test.

To determine whether a decrease in DUSP1 contributes to the activation of p38 MAPK pathway in FOXM1-deficient macrophages, we overexpressed DUSP1 in *siFoxm1*-transfected RAW 264.7 cells. The efficient overexpression of DUSP1 is shown by increased *Dusp1* mRNA (Fig 6A). Inhibition of FOXM1 increased the expression of p38 MAPK downstream targets *Tnf*, *Il6* and *Il1b* in macrophages (Fig 6A), a finding consistent with the *in vivo* data (Fig 4A and 4B). Overexpression of DUSP1 was sufficient to prevent the increase of *Tnf*, *Il6* and *Il1b* mRNAs in FOXM1-deficient macrophages (Fig 6A) and to decrease phospho-p38 MAPK protein in FOXM1-deficient macrophages (Fig 6B). While supernatant from FOXM1-deficient macrophages increased number of 3T3 fibroblasts and their activation, over-expression of DUSP1 attenuated these effects (Fig 6C and 6D), supporting a critical role of DUSP1 in pro-fibrotic signaling from macrophages to fibroblasts. Altogether, these data indicate that FOXM1 inhibits activation of p38 MAPK pathway and expression of its targets through transcriptional activation of DUSP1.

### Adoptive transfer of wild type macrophages protects my*Foxm1*^-/-^ mice from bleomycin-induced pulmonary fibrosis

Next, we tested whether adoptive transfer of wild type (WT) macrophages decreases the severe fibrotic burden in bleomycin-treated my*Foxm1*^-/-^ mice. Control and my*Foxm1*^-/-^ mice were treated twice with 1.0 U/Kg bleomycin via intra-tracheal instillation followed by adoptive transfer of bone marrow-derived WT monocytic progenitors (Fig 6E). The efficiency of the adoptive transfer was shown by the presence of FOXM1-positive macrophages in bleomycin-treated my*Foxm1*^-/-^ lungs (S8 Fig). FOXM1-positive macrophages were absent in bleomycin-treated my*Foxm1*^-/-^ lungs without adoptive transfer (S8 Fig). Lung histology was evaluated, and collagen depositions were measured to assess fibrosis after the adoptive transfer. Adoptive transfer of WT monocytes into bleomycin-treated my*Foxm1*^-/-^ mice significantly reduced the fibrotic load to collagen levels seen in control mice (Fig 6F and 6G). Thus, adoptive transfer of WT macrophages decreases pulmonary fibrosis in my*Foxm1*^-/-^ mice. Altogether, FOXM1 expression in macrophages is critical to prevent pulmonary fibrosis.

## Discussion

Pulmonary fibrosis results from deregulated repair of damaged tissue and is refractory to available treatment. Several different cell types are essential for the development of pulmonary fibrosis, including epithelial cells (AECII), fibroblasts and inflammatory cells [31]. The Forkhead box M1 (FOXM1) is a transcription factor that is highly expressed during embryogenesis, but is not expressed in quiescent adult lung [19]. FOXM1 stimulates cellular proliferation and its expression is induced in lung cancers [32, 33]. High levels of FOXM1 were found in tumor cells and tumor-associated macrophages [24, 25]. Genetic deletion of *Foxm1* in macrophages inhibited lung cancer. FOXM1-deficient macrophages failed to migrate to the lung tissue resulting in reduced tumor-associated inflammation known to promote tumorigenesis [24]. Thus, FOXM1 has oncogenic and pro-inflammatory role in adult lungs during carcinogenesis. In addition to lung cancer, it was also shown that FOXM1 is highly expressed in several other chronic lung diseases, including COPD, asthma and IPF [20–22, 34, 35]. In human and mouse fibrotic lungs, increased levels of FOXM1 were found in AECII and fibroblasts [20, 21]. Mouse genetic studies demonstrated that deletion of *Foxm1* from either AECII [20] or fibroblasts [21] inhibits pulmonary fibrosis. Likewise, overexpression of FOXM1 in AECII exacerbated pulmonary fibrosis induced by thoracic irradiation or bleomycin [20], which is consistent with pro-fibrotic role of FOXM1. These studies suggest that inhibition of FOXM1 can be beneficial for the treatment of IPF and other fibrotic diseases. In the present manuscript, we found that in addition to epithelial cells and fibroblasts, FOXM1 is highly overexpressed in pulmonary macrophages and neutrophils of mouse and human fibrotic lungs. Surprisingly, deletion of *Foxm1* from myeloid cells exacerbated bleomycin-induced pulmonary fibrosis, indicating anti-fibrotic role of FOXM1 in myeloid lineage. Thus, FOXM1 can induce or inhibit fibrosis depending on cell specificity and therefore, the use of FOXM1 inhibitors in IPF should be approached with caution. Interestingly, *Mlyz-Cre* deleted *Foxm1* from all myeloid cells [34]. It is possible that the macrophage lineage is central to the fibrotic phenotype in my*Foxm1*^-/-^ mice. Adoptive transfer of monocytic cells was sufficient to decrease fibrosis in my*Foxm1*^-/-^ lungs, providing a direct support to this concept.

We have previously showed that FOXM1-deficient macrophages fail to migrate to the lung tissue during lung tumorigenesis [24]. After acute liver injury, deletion of *Foxm1* from macrophages severely reduced macrophage recruitment to the injured liver and delayed liver repair [25]. FOXM1 regulated migration of macrophages through transcriptional activation of chemokine receptors *Cx3cr1* and *Ccr2* [24, 25]. Surprisingly, in the present studies we found that deletion of *Foxm1* did not affect the numbers of macrophages after bleomycin lung injury. Moreover, expression of *Cx3cr1* and *Ccr2* in macrophages were not changed after deletion of *Foxm1*. Thus, FOXM1 activates *Cx3cr1* and *Ccr2* in macrophages during lung cancer and acute liver injury/repair [24, 25], but is not doing so during lung fibrosis. These surprising results indicate that functions of FOXM1 in macrophages are dependent on type of the injury and tissue/organ specificity. Being a transcription factor, FOXM1 physically binds to other co-activators/co-repressors. It is possible that specific combinations of these regulatory co-factors alter FOXM1 functions by activating different sets of transcription targets. In addition, transcription factors are often regulated by the epigenetic landscape. Thus, co-activators/co-repressors and epigenetic changes in macrophages could explain distinct functions of FOXM1 during lung cancer, acute injury and lung fibrosis.

Mitogen-activated protein kinase (MAPK) superfamily plays a crucial role in growth, differentiation, and response to stress [36]. The p38 MAPK pathway is activated by environmental stresses, and plays a central role in inflammatory response in the lung. Activation of the p38 MAPK signaling cascade induces secretion of IL-8 by bronchial epithelial cells [37], increases expression of ICAM-1 adhesion molecule on endothelial cells [38], stimulates secretion of TNF-α, IL-6 and IL-8 by neutrophils [39] and monocytes [40]. Activation of the p38 MAPK pathway directly correlates with severity of pulmonary fibrosis. In comparison to normal lung, p38 MAPK pathway is activated in epithelial, endothelial, smooth muscle cells, and fibroblasts in lung tissues from patients with IPF [41]. Activation of p38 MAPK pathway by TGF-β is critical for aberrant proliferation of pulmonary interstitial fibroblasts [42]. P38 MAPK activates MK2 and induces differentiation of fibroblasts into myofibroblasts [43]. On the other hand, inhibition of p38 MAPK signaling decreased expression of collagen I and fibronectin [44]. Inhibition of p38 MAPK signaling with specific inhibitor FR-167653 prevented bleomycin-induced pulmonary fibrosis in mice [30], indicating an essential role of p38 MAPK pathway in pulmonary fibrotic responses. The important contribution of the current study is that FOXM1 inhibits p38 MAPK signaling pathway in macrophages through transcriptional activation of *Dusp1*, a negative regulator of p38 MAPK. In the absence of FOXM1, the p38 MAPK pathway is activated in macrophages leading to production of pro-fibrotic mediators IL-1β, IL-6 and TNF-α that directly stimulate fibroblast activation and survival, exacerbating pulmonary fibrosis. Given a robust pro-fibrotic activity of FOXM1 in macrophage lineage and critical role of macrophages in mouse models of pulmonary fibrosis[45],[46],[47, 48], the use of FOXM1 inhibitors in IPF or other fibrotic diseases should be approached with caution.

In summary, macrophage-specific inactivation of FOXM1, exacerbates bleomycin-induced pulmonary fibrosis. FOXM1 inhibits the p38 MAPK signaling pathway and its downstream target genes, TNFα, IL-6 and IL-1β, through transcriptional activation of *Dusp1*. Our studies suggest an important limitation of using pharmacological FOXM1 inhibitors in IPF.

## Materials and methods

### Transgenic mice and bleomycin-induced fibrosis model

*Foxm1* was deleted from macrophages by breeding *Foxm1*^fl/fl^ mice [25] with *LysM-Cre*^tg/-^ transgenic mice (The Jackson Laboratory, Bar Harbor, ME, USA) to produce *LysM-Cre*^tg/-^: *Foxm1*^fl/fl^ mice (my*Foxm1*^-/-^) as previously described [24, 25]. Single transgenic *Foxm1*^fl/fl^ littermates were used as controls. Animal studies were reviewed and approved by the Animal Care and Use Committee of Cincinnati Children’s Hospital Research Foundation. Pulmonary fibrosis was induced with bleomycin sulfate (EMD Millipore, Billerica, MA) [20]. 1.0 U / Kg of bleomycin was administered intratracheally once per week for three consecutive weeks. For the time-course experiment, CD57Bl/6 mice were administered 1.0 U / Kg of bleomycin intratracheally once per week for 4 weeks. Mice were sacrificed one week after final bleomycin treatment. Quantification of fibrosis was determined by measuring lung collagen content using the Sircol collagen assay (Biocolor, Carrickfergus, UK). Total RNA was prepared from BALF cells, and flow-sorted alveolar and interstitial macrophages from bleomycin-treated my*Foxm1*^-/-^ or control mice. mRNA levels of specific genes were measured by qRT-PCR using TaqMan probes (S1 Table) and the StepOnePlus Real-Time PCR system (Applied Biosystems) as previously described [49].

### Human Lung samples

Lung samples were obtained at the University of Vienna and the University of Giessen Lung Center from patients with IPF (n = 9) and control donors (n = 6) anonymously following lung transplantation. Diagnosis was done according to the American Thoracic Society/European Respiratory Society (ATS/ERS) criteria for IPF. Utilization of human lung samples was reviewed and approved by the Ethics Committee of Justus-Liebig-University of Giessen. Fibrotic lesions were identified based on the histology of the lung tissue sections [50, 51].

### Immunohistochemistry and immunofluorescence

Mouse lungs were inflated, fixed and embedded in paraffin. Sections (5 μm) were stained with hematoxylin and eosin (H&E) to assess gross lung morphology. Immunohistochemical staining and immunofluorescence were performed as described previously [52]. The list of antibodies is presented in S2 Table. Antibody-antigen complexes were detected using biotinylated secondary antibodies followed by avidin-biotin-horseradish peroxidase complex and 3,3’-diaminobenzidine substrate (Vector Labs), as previously described [53],[54]. Sections were counterstained with nuclear fast red (Vector Labs). For immunofluorescence imaging, secondary antibodies conjugated with Alexa Fluor 488 or Alexa Fluor 594 (Invitrogen/Molecular Probes) were used. Nuclei were counterstained with DAPI. Fibrosis was visualized using Masson’s trichrome staining (Siemens) and Sirius Red Fast Green staining (Chondrex). Images were obtained using a Zeiss AxioPlan2 microscope.

### Flow cytometry

Inflammatory cells were analyzed from bleomycin-treated lungs of my*Foxm1*^-/-^ and *Foxm1*^fl/fl^ mice by flow cytometry as described previously [55]. The following antibodies were used to stain inflammatory cells: anti-F4/80 (clone BM8; eBiosciences), anti-CD11b (clone M1/70; eBiosciences), anti-Ly-6C (clone HK1.4; BioLegend), anti–Ly-6G (clone 1A8; BioLegend), anti-CD68 (clone FA-11; Biolegend), anti-CD45 (clone 30-F11; BD Pharmingen), anti-CD3 (Clone 17A2; eBioscience), anti-B220 (Clone RA3-6B2; eBioscience). Dead cells were excluded using Fixable viability dye APC-eF780 (eBioscience). Stained cells were analyzed using a BD LSR II flow cytometer. Stained cells were separated using cell sorting (five-laser FACSAria II; BD Biosciences). Specific cell subsets were identified using the indicated surface marker phenotypes: neutrophils, CD45^+^ CD11b^+^ Ly6G^+^; monocytes, CD45^+^ CD11b^+^ Ly6G^−^ F4/80^low^; interstitial macrophages, CD45^+^ CD11b^+^ Ly6G^−^ F4/80^high^ CD68^low^; and alveolar macrophages, CD45^+^ CD11b^+^ Ly6G^−^ F4/80^high^ CD68^high^; T cells, CD45^+^ CD3^+^; B cells, CD45^+^ B220^+^ [26, 34, 56].

### Cell culture, qRT-PCR and Western blot

The *Foxm1* siRNA (Dharmacon) was used to transiently knockdown *Foxm1* in mouse macrophage cell line RAW 264.7 (ATCC) using Dharmafect Duo transfection reagent (Dharmacon) according to the manufacturer’s instructions. Non-targeting siRNA was used as control. Cells were collected for RNA and protein extraction 48 hours after transfection. Culture supernatant was collected for 3T3 proliferation assay. For *in vitro* cytokine-neutralization assay, supernatant from RAW 264.7 cells was incubated with neutralizing antibodies against IL-6 (0.03μg/mL), IL-1β (0.25μg/mL), or TNF-α (0.1μg/mL) at 37°C for 2 hours. *In vitro* rescue experiments were performed by dual transfection of si*Foxm1* and pHIV*Dusp1* overexpression plasmids using Dharmafect Duo transfection reagent (Dharmacon). Empty plasmid and non-targeting siRNA were used as control. Cells were collected for RNA and protein extraction 48 hours after transfection. Culture supernatant from macrophages was collected for 3T3 proliferation assay. 3T3 cells were cultured in the collected supernatant for 48 hours and number of live cells were counted using trypan blue by automated cell counter Countess^TM^ II FL (Life Technologies). Protein and mRNA from 3T3 cells were used for qRT-PCR and Western blot as previously described[33]. TaqMan probes are provided in S1 Table. The following antibodies were used: FOXM1 (1:500, C-20, Santa Cruz), DUSP1 (1:1000, Abcam), phos-p38 MAPK (1:500, Cell Signaling Technology), total p38 MAPK (1:1000, Abcam), IL-6 (1:500, R&D), IL-1β (1:500, R&D), TNF-α (1:500, Abcam), β-ACTIN (1:1000, Santa Cruz).

### Chromatin immunoprecipitation assay

RAW264.7 mouse macrophages were cultured *in vitro* according to the ATCC protocol. Cells were cross-linked by addition of formaldehyde, sonicated, and used for immunoprecipitation using a FOXM1 rabbit polyclonal antibody (C-20; Santa Cruz Biotechnology, Santa Cruz, CA, USA). Reverse cross-linked chromatin immune-precipitation DNA samples were subjected to qRT-PCR using EvaGreen dye and the following PCR primers (5’ to 3’): m*Dusp1* -1kb Forward GTTAGTAGGAGTCCGGGTCCA + Reverse GCCAACAAGCTCTTCCGTGC; m*Dusp1* -100bp: Forward ATCTGGGCTCAGCTGTTCTG + Reverse TTCACTCTGGCCCATGAAGC. Binding of FOXM1 was normalized to the DNA of the samples immunoprecipitated with isotype control IgG.

### Cloning of the mouse *Dusp1* promoter region and luciferase assay.

The mouse *Dusp1* promoter corresponding to the -1033bp to -17bp was PCR amplified from C57/B6 gDNA using the following primers (5’ to 3’): Fwd. GAG GTAC CAG GAG GAT ATA GAA AGC GGC; Rev. ATCTCGAGTTCGGGGAGATGATACTCC. The 1.0kb promoter region was cloned into the KpnI and XhoI sites of the pGL3 luciferase reporter plasmid (Promega). Dual luciferase reporter assay was performed using the Dusp1 luciferase reporter with CMV-empty or CMV-*FOXM1* overexpression plasmids.

### Generation of mouse Dusp1 overexpression plasmid

Mouse cDNA was PCR amplified using *Dusp1* specific primers: Forward: 5’-TGATTCTAGAATGGTGATGGAGGTGGGCA-3’, Reverse: 5’- TGGATCCTCAAGCGTAATCTGGAACATCGTATGGGTAGCTTGGAGAGGTGGTGA-3’. *Dusp1* cDNA was inserted into pHIV-EGFP plasmid (Addgene) digested with BamHI and XbaI. Plasmid was sequenced to confirm insertion.

### Isolation and adoptive transfer of monocytes during bleomycin-induced lung injury

For adoptive transfer experiments, recipient *Foxm1*^fl/fl^ and my*Foxm1*^-/-^ mice were treated with 1.0 U / Kg bleomycin twice via intratracheal administrations. Monocytes were isolated from the bone marrow of donor *Foxm1*^fl/fl^ or my*Foxm1*^-/-^ mice as described [25, 57]. Cells were incubated with a biotin-conjugated anti-CD115 (M-CSF receptor) antibody (eBioscience) followed by anti-biotin MACS beads (Miltenyi Biotech, Auburn, CA, USA). A positive cell fraction was obtained through magnetic separation and labeled with CFSE as described [57]. The percentage of monocytes in the positive fraction was 85% as determined by flow cytometry defining the monocytic phenotype as CD115^+^/CD11b^+^/Ly-6G^−^/Ly-6C^hi^/F4/80^lo^ cells. One week after treatment with bleomycin, 5 × 10^5^ labeled cells were injected into tail veins of my*Foxm1*^-/-^ or control *Foxm1*^fl/f*l*^ mice. Recipient mice were harvested one week after adoptive transfer and fibrotic load was assessed.

### Generation of Bone-marrow derived macrophages and Collagen degradation assay

Bone marrow derived macrophages were generated as previously described [58]. Bone marrow cells were obtained from 8–10 weeks-old *Foxm1*^fl/fl^, or my*Foxm1*^-/-^ mice. Mononuclear cells were isolated and cultured overnight. The following day, non-adherent cells were recovered, transferred to a new dish and cultured in in DMEM containing 10% heat-inactivated FBS, 100 U/ml penicillin, 100 μg/ml streptomycin, 0.25 μg/ml Amphotericin B, 10ng/ml GM-CSF and 5 ng/ml M-CSF (both from R&D Systems) to allow differentiation and expansion of macrophages. After 48 hours, the culture medium was changed, and adherent bone marrow-derived macrophages were isolated by trypsin-EDTA 5 days after seeding. Cells were plated at a concentration of 50,000 cells per well in black 96-well plates in triplicate in DMEM containing 10% heat-inactivated FBS, 100 U/ml penicillin, 100 μg/ml streptomycin, 0.25 μg/ml Amphotericin B, and 10ng/mL LPS and cultured overnight at 37°C in a humidified environment containing 5% CO_2_. Following day, cells were washed with PBS and incubated in PBS with 1μg/ml of FITC-conjugated type I collagen (Invitrogen) in a total volume of 100 μl. After 4 hours, fluorescence from cleaved collagen was quantified using a spectrofluorometer (SpectraMax Minimax 300) at 499nm Absorption maximum and 525nm Emission maximum. The fluorescent signal from control wells containing only collagen was subtracted from that from wells containing macrophages and collagen.

### Statistical analysis

Statistical significance differences in measured variables between experimental and control groups were assessed by Student’s *t*-test (2-tailed) *P*-values <0.05 were considered significant. Values for all measurements were expressed as the mean ± standard deviation or as the mean ± standard error of mean. Statistical analysis was performed, and data were graphically displayed using GraphPad Prism v.5.0 for Windows (GraphPad Software, Inc.).

### Study approval

All animal studies were approved by Cincinnati Children’s Research Foundation Institutional Animal Care and Use Committee (protocol IACUC2016-0070). The Cincinnati Children’s Research Foundation Institutional Animal Care and Use Committee is an AAALAC and NIH accredited institution (NIH Insurance #8310801).

## Supporting information

S1 Fig Fibrotic foci in the IPF lung. Fibrotic lesions in IPF patient lung sections were identified based on the characteristic histology using H&E staining (top panel). Immunostaining shows the presence of activated myofibroblasts positive for αSMA (green, bottom left panel) and the presence of CD68^+^ macrophages (red) in the fibrotic lesions of IPF lungs.
(TIF)

S2 Fig Bleomycin treatment increased *Foxm1* mRNA in lung neutrophils. Neutrophils were isolated from bleomycin-treated or saline-treated WT lungs using Ly6G magnetic beads. qRT-PCR was used to measure *Foxm1* mRNA. N = 8 mice per group. *Actb* mRNA was used for normalization. *** = *P* < 0.001, by Student’s t-test.
(TIF)

S3 Fig Conditional deletion of *Foxm1* in myeloid cells exacerbates bleomycin-induced pulmonary fibrosis in mice. 8–10 weeks old my*Foxm1*^-/-^ and *Foxm1*^fl/fl^ mice were treated with bleomycin (1U/kg; once a week for three weeks). Mice were sacrificed, and lungs were collected one week after the final bleomycin treatment. H&E staining of lungs of 3 individual mice per group shows increased pulmonary fibrosis in bleomycin-treated my*Foxm1*^-/-^ mice (bottom panels) compared to control bleomycin-treated mice (top panels). Scale bar = 200*μ*m.
(TIF)

S4 Fig Deletion of *Foxm1* from myeloid cells does not affect the number of immune cells in the lungs of bleomycin-treated mice. **(A)** Lung cell suspensions from bleomycin-treated mice were used for flow cytometry. Representative gating strategy for flow cytometry is shown. Single, live cells were gated to identify neutrophils (CD45^+^ CD11b^+^ Ly6G^+^), monocytes (CD45^+^ CD11b^+^ Ly6G^-^ F4/80^low^), alveolar macrophages (CD45^+^ CD11b^+^ Ly6G^-^ F4/80^hi^ CD68^hi^), interstitial macrophages (CD45^+^ CD11b^+^ Ly6G^-^ F4/80^hi^ CD68^low^), B cells (CD45^+^ B220^+^), and T cells (CD45^+^ CD3^+^). **(B)** No difference in *Cx3cr1* and *Ccr2* mRNAs was found in interstitial macrophages FACS-sorted from bleomycin treated my*Foxm1*^-/-^ mice compared to controls. qRT-PCR was used to measure mRNAs. *Actb* mRNA was used for normalization. N = 3 mice per group. **(C)** No difference was found in the number of T cells in bleomycin treated my*Foxm1*^-/-^ lungs compared to controls. Number of CD3^+^ T cells were counted in 5 random fields and presented as mean ± SEM (n = 3 mice per group). Scale bar = 50*μ*m.
(TIF)

S5 Fig FOXM1-deficient macrophages express decreased anti- fibrotic marker ARG1. **(A)** Decreased expression of ARG1 is found in bleomycin-treated lungs of my*Foxm1*^-/-^ mice compared to controls. Number of ARG1^+^ cells were counted in 5 random fields and presented as mean ± SEM (n = 3 mice per group). Scale bar = 200*μ*m. **(B)** *Arg1* mRNA is decreased in BALF cells isolated from bleomycin-treated my*Foxm1*^-/-^ mice compared to controls as determined by qRT-PCR. *Actb* mRNA was used for normalization (n = 3 mice per group). **(C)** *Arg1* mRNA is decreased in interstitial macrophages FACS-sorted from bleomycin-treated my*Foxm1*^-/-^ mice compared to controls as determined by qRT-PCR. *Actb* mRNA was used for normalization (n = 3 mice per group). * = *P >* 0.05; ** = *P* > 0.01, *** = *P* < 0.001, by Student’s t-test.
(TIF)

S6 Fig FOXM1-deficient macrophages have decreased expression of *Mrc1* and inefficiently degrade collagen. **(A)** *Mrc1* mRNA is decreased in interstitial macrophages FACS-sorted from bleomycin-treated my*Foxm1*^-/-^ mice compared to controls as determined by qRT-PCR. *Actb* mRNA was used for normalization. N = 3 mice per group. **(B)** *Mrc1* mRNA is decreased in BALF cells isolated from bleomycin-treated my*Foxm1*^-/-^ mice compared to controls as determined by qRT-PCR. *Actb* mRNA was used for normalization. N = 3 mice per group. **(C)** *Tgfb1* mRNA is increased in total lung RNA from bleomycin-treated *myFoxm1*^*-/-*^ mice as determined by qRT-PCR. *Actb* mRNA was used for normalization (n = 5 mice per group). **(D)** Depletion of *Foxm1* in mouse macrophages did not change *Tgfb1* mRNA as shown by qRT-PCR. RAW264.7 cells were transfected with control siRNA or si*Foxm1* and mRNA levels were measured by qRT-PCR. *Actb* mRNA was used for normalization (n = 3). **(E)** *Foxm1* mRNA is decreased in bone-marrow derived macrophages from my*Foxm1*^-/-^ mice compared to controls as determined by qRT-PCR. *Actb* mRNA was used for normalization. N = 3 mice per group. **(F)** *Csfr1*, *Csf3*, and *Cxcr4* mRNA levels are unchanged in bone-marrow derived macrophages from treated my*Foxm1*^-/-^ mice as compared to control mice as determined by qRT-PCR. *Actb* mRNA was used for normalization (n = 3 mice per group). **(G)** Collagen degradation is decreased in bone-marrow derived macrophages from my*Foxm1*^-/-^ mice compared to controls. The fluorescent signal from control wells containing only collagen was subtracted from that from wells containing macrophages and collagen. Assay was done in triplicates using bone-marrow derived macrophages from 3 individual mice per group. * = *P >* 0.05; ** = *P* > 0.01, *** = *P* < 0.001, by Student’s t-test.
(TIF)

S7 Fig **(A)** Supernatant from *Foxm1*-deficient macrophages decreases fibroblast survival through IL-6, IL-1β, and TNF-α. The NIH-3T3 fibroblasts were cultured in supernatant obtained from RAW264.7 macrophages transfected with control siRNA or si*Foxm1*. IL-6, IL-1β, and TNF-α were inhibited in the cell culture using neutralizing Abs. 48 hours after addition of supernatant, the numbers of live fibroblasts were counted using trypan blue (n = 3). Neutralizing antibody against IgG was used as control. **(B-C)** Supernatant from *Foxm1*-deficient macrophages decreases *Acta2* and *Col1a1* mRNAs in fibroblasts through IL-6 and IL-1β as shown by qRT-PCR. mRNA levels in fibroblasts were analyzed by qRT-PCR. *Actb* mRNA was used for normalization (n = 3).
(TIF)

S8 Fig Adoptive transfer of wild-type macrophages increases the number of FOXM1-positive macrophages in my*Foxm1*^-/-^ lungs. Immunostaining shows FOXM1-positive macrophages in bleomycin-treated control lungs (top panels) and my*Foxm1*^-/-^ lungs (bottom panels) after adoptive transfer of WT monocytes. No FOXM1-positive macrophages are found in bleomycin-treated my*Foxm1*^-/-^ lungs without adoptive transfer (middle panels). Control and my*Foxm1*^-/-^ mice were treated twice weekly with bleomycin (1.0 U / Kg). One week after treatment, bone marrow-derived monocytes were injected (I.V.) into both groups of mice. Mice were sacrificed one week after the adoptive transfer. Numbers of FOXM1^+^ cells were counted in 5 random fields and presented as mean ± SEM. N = 3 mice per group.
(TIF)

S1 Table Taqman probes. (DOCX)

S2 Table Antibodies. (DOCX)

S1 Data (XLSX)

## References

[1] Murray PJ, Wynn TA. Protective and pathogenic functions of macrophage subsets. Nat Rev Immunol. 2011;11(11):723–37. 10.1038/nri3073 21997792 PMC3422549

[2] Wynn TA. Cellular and molecular mechanisms of fibrosis. J Pathol. 2008;214(2):199–210. 10.1002/path.2277 18161745 PMC2693329

[3] Wynn TA. Fibrotic disease and the T(H)1/T(H)2 paradigm. Nat Rev Immunol. 2004;4(8):583–94. 10.1038/nri1412 15286725 PMC2702150

[4] Xiao W, Hong H, Kawakami Y, Lowell CA, Kawakami T. Regulation of myeloproliferation and M2 macrophage programming in mice by Lyn/Hck, SHIP, and Stat5. J Clin Invest. 2008;118(3):924–34. 10.1172/JCI34013 18246197 PMC2214849

[5] Gabbiani G. The myofibroblast in wound healing and fibrocontractive diseases. J Pathol. 2003;200(4):500–3. 10.1002/path.1427 12845617

[6] Pesce JT, Ramalingam TR, Wilson MS, Mentink-Kane MM, Thompson RW, Cheever AW, et al Retnla (relmalpha/fizz1) suppresses helminth-induced Th2-type immunity. PLoS Pathog. 2009;5(4):e1000393 10.1371/journal.ppat.1000393 19381262 PMC2663845

[7] Wilson MS, Elnekave E, Mentink-Kane MM, Hodges MG, Pesce JT, Ramalingam TR, et al IL-13Ralpha2 and IL-10 coordinately suppress airway inflammation, airway-hyperreactivity, and fibrosis in mice. J Clin Invest. 2007;117(10):2941–51. 10.1172/JCI31546 17885690 PMC1978425

[8] Pesce JT, Ramalingam TR, Mentink-Kane MM, Wilson MS, El Kasmi KC, Smith AM, et al Arginase-1-expressing macrophages suppress Th2 cytokine-driven inflammation and fibrosis. PLoS Pathog. 2009;5(4):e1000371 10.1371/journal.ppat.1000371 19360123 PMC2660425

[9] Cabrera S, Gaxiola M, Arreola JL, Ramirez R, Jara P, D'Armiento J, et al Overexpression of MMP9 in macrophages attenuates pulmonary fibrosis induced by bleomycin. Int J Biochem Cell Biol. 2007;39(12):2324–38. 10.1016/j.biocel.2007.06.022 17702637

[10] Fallowfield JA, Mizuno M, Kendall TJ, Constandinou CM, Benyon RC, Duffield JS, et al Scar-associated macrophages are a major source of hepatic matrix metalloproteinase-13 and facilitate the resolution of murine hepatic fibrosis. J Immunol. 2007;178(8):5288–95. 10.4049/jimmunol.178.8.5288 17404313

[11] Barron L, Wynn TA. Fibrosis is regulated by Th2 and Th17 responses and by dynamic interactions between fibroblasts and macrophages. Am J Physiol Gastrointest Liver Physiol. 2011;300(5):G723–8. 10.1152/ajpgi.00414.2010 21292997 PMC3302189

[12] Roberts AB, Sporn MB, Assoian RK, Smith JM, Roche NS, Wakefield LM, et al Transforming growth factor type beta: rapid induction of fibrosis and angiogenesis in vivo and stimulation of collagen formation in vitro. Proc Natl Acad Sci U S A. 1986;83(12):4167–71. 10.1073/pnas.83.12.4167 2424019 PMC323692

[13] Kolb M, Margetts PJ, Anthony DC, Pitossi F, Gauldie J. Transient expression of IL-1beta induces acute lung injury and chronic repair leading to pulmonary fibrosis. J Clin Invest. 2001;107(12):1529–36. 10.1172/JCI12568 11413160 PMC200196

[14] Shimokado K, Raines EW, Madtes DK, Barrett TB, Benditt EP, Ross R. A significant part of macrophage-derived growth factor consists of at least two forms of PDGF. Cell. 1985;43(1):277–86. 10.1016/0092-8674(85)90033-9 2416458

[15] Nagaoka I, Trapnell BC, Crystal RG. Upregulation of platelet-derived growth factor-A and -B gene expression in alveolar macrophages of individuals with idiopathic pulmonary fibrosis. J Clin Invest. 1990;85(6):2023–7. 10.1172/JCI114669 2347924 PMC296674

[16] Broekelmann TJ, Limper AH, Colby TV, McDonald JA. Transforming growth factor beta 1 is present at sites of extracellular matrix gene expression in human pulmonary fibrosis. Proc Natl Acad Sci U S A. 1991;88(15):6642–6. 10.1073/pnas.88.15.6642 1862087 PMC52144

[17] Byrne AJ, Maher TM, Lloyd CM. Pulmonary Macrophages: A New Therapeutic Pathway in Fibrosing Lung Disease? Trends Mol Med. 2016. 10.1016/j.molmed.2016.02.004 26979628

[18] Vittal R, Fisher A, Gu H, Mickler EA, Panitch A, Lander C, et al Peptide-mediated inhibition of mitogen-activated protein kinase-activated protein kinase-2 ameliorates bleomycin-induced pulmonary fibrosis. Am J Respir Cell Mol Biol. 2013;49(1):47–57. 10.1165/rcmb.2012-0389OC 23470623 PMC3727887

[19] Kalin TV, Ustiyan V, Kalinichenko VV. Multiple faces of FoxM1 transcription factor: lessons from transgenic mouse models. Cell Cycle. 2011;10(3):396–405. 10.4161/cc.10.3.14709 21270518 PMC3115014

[20] Balli D, Ustiyan V, Zhang Y, Wang IC, Masino AJ, Ren X, et al Foxm1 transcription factor is required for lung fibrosis and epithelial-to-mesenchymal transition. EMBO J. 2013;32(2):231–44. 10.1038/emboj.2012.336 23288041 PMC3553386

[21] Penke LR, Speth JM, Dommeti VL, White ES, Bergin IL, Peters-Golden M. FOXM1 is a critical driver of lung fibroblast activation and fibrogenesis. J Clin Invest. 2018;128(6):2389–405. 10.1172/JCI87631 29733296 PMC5983327

[22] Im J, Lawrence J, Seelig D, Nho RS. FoxM1-dependent RAD51 and BRCA2 signaling protects idiopathic pulmonary fibrosis fibroblasts from radiation-induced cell death. Cell Death Dis. 2018;9(6):584 10.1038/s41419-018-0652-4 29789556 PMC5964221

[23] Franklin CC, Kraft AS. Conditional expression of the mitogen-activated protein kinase (MAPK) phosphatase MKP-1 preferentially inhibits p38 MAPK and stress-activated protein kinase in U937 cells. J Biol Chem. 1997;272(27):16917–23. 10.1074/jbc.272.27.16917 9202001

[24] Balli D, Ren X, Chou FS, Cross E, Zhang Y, Kalinichenko VV, et al Foxm1 transcription factor is required for macrophage migration during lung inflammation and tumor formation. Oncogene. 2012;31(34):3875–88. 10.1038/onc.2011.549 22139074 PMC3297705

[25] Ren X, Zhang Y, Snyder J, Cross ER, Shah TA, Kalin TV, et al Forkhead box M1 transcription factor is required for macrophage recruitment during liver repair. Mol Cell Biol. 2010;30(22):5381–93. 10.1128/MCB.00876-10 20837707 PMC2976366

[26] Ren X. Forkhead Box M1 Transcription Factor Is Required for Macrophage Recruitment during Liver Repair. Mol Cell Biol. 2010. 10.1128/MCB.00876-10 PMC2976366 20837707

[27] Zhang J, Patel JM. Role of the CX3CL1-CX3CR1 axis in chronic inflammatory lung diseases. Int J Clin Exp Med. 2010;3(3):233–44. 20827321 PMC2929949

[28] Madsen DH, Leonard D, Masedunskas A, Moyer A, Jurgensen HJ, Peters DE, et al M2-like macrophages are responsible for collagen degradation through a mannose receptor-mediated pathway. J Cell Biol. 2013;202(6):951–66. 10.1083/jcb.201301081 24019537 PMC3776354

[29] Qian F, Deng J, Wang G, Ye RD, Christman JW. Pivotal Role of Mitogen-Activated Protein Kinase-Activated Protein Kinase 2 in Inflammatory Pulmonary Diseases. Curr Protein Pept Sci. 2016;17(4):332–42. 10.2174/1389203716666150629121324 26119506 PMC4878395

[30] Matsuoka H, Arai T, Mori M, Goya S, Kida H, Morishita H, et al A p38 MAPK inhibitor, FR-167653, ameliorates murine bleomycin-induced pulmonary fibrosis. Am J Physiol Lung Cell Mol Physiol. 2002;283(1):L103–12. 10.1152/ajplung.00187.2001 12060566

[31] Wynn TA. Integrating mechanisms of pulmonary fibrosis. J Exp Med. 2011;208(7):1339–50. 10.1084/jem.20110551 21727191 PMC3136685

[32] Kalinichenko VV, Kalin TV. Is there potential to target FOXM1 for 'undruggable' lung cancers? Expert Opin Ther Targets. 2015;19(7):865–7. 10.1517/14728222.2015.1042366 25936405 PMC4836176

[33] Milewski D, Balli D, Ustiyan V, Le T, Dienemann H, Warth A, et al FOXM1 activates AGR2 and causes progression of lung adenomas into invasive mucinous adenocarcinomas. PLoS Genet. 2017;13(12):e1007097 10.1371/journal.pgen.1007097 29267283 PMC5755924

[34] Ren X, Shah TA, Ustiyan V, Zhang Y, Shinn J, Chen G, et al FOXM1 promotes allergen-induced goblet cell metaplasia and pulmonary inflammation. Mol Cell Biol. 2013;33(2):371–86. 10.1128/MCB.00934-12 23149934 PMC3554115

[35] Sun L, Ren X, Wang IC, Pradhan A, Zhang Y, Flood HM, et al The FOXM1 inhibitor RCM-1 suppresses goblet cell metaplasia and prevents IL-13 and STAT6 signaling in allergen-exposed mice. Sci Signal. 2017;10(475). 10.1126/scisignal.aai8583 28420758

[36] Cobb MH, Goldsmith EJ. How MAP kinases are regulated. J Biol Chem. 1995;270(25):14843–6. 10.1074/jbc.270.25.14843 7797459

[37] Matsumoto K, Hashimoto S, Gon Y, Nakayama T, Horie T. Proinflammatory cytokine-induced and chemical mediator-induced IL-8 expression in human bronchial epithelial cells through p38 mitogen-activated protein kinase-dependent pathway. J Allergy Clin Immunol. 1998;101(6 Pt 1):825–31. 10.1016/S0091-6749(98)70311-2 9648711

[38] Jersmann HPA, Hii CST, Ferrante JV, Ferrante A. Bacterial Lipopolysaccharide and Tumor Necrosis Factor Alpha Synergistically Increase Expression of Human Endothelial Adhesion Molecules through Activation of NF-κB and p38 Mitogen-Activated Protein Kinase Signaling Pathways. Infect Immun. 2001;69(3):1273–9. 10.1128/IAI.69.3.1273-1279.2001 11179288 PMC98017

[39] Zu YL, Qi J, Gilchrist A, Fernandez GA, Vazquez-Abad D, Kreutzer DL, et al p38 mitogen-activated protein kinase activation is required for human neutrophil function triggered by TNF-alpha or FMLP stimulation. J Immunol. 1998;160(4):1982–9. 9469462

[40] Manthey CL, Wang SW, Kinney SD, Yao Z. SB202190, a selective inhibitor of p38 mitogen-activated protein kinase, is a powerful regulator of LPS-induced mRNAs in monocytes. J Leukoc Biol. 1998;64(3):409–17. 10.1002/jlb.64.3.409 9738669

[41] Yoshida K, Kuwano K, Hagimoto N, Watanabe K, Matsuba T, Fujita M, et al MAP kinase activation and apoptosis in lung tissues from patients with idiopathic pulmonary fibrosis. J Pathol. 2002;198(3):388–96. 10.1002/path.1208 12375272

[42] Khalil N, Xu YD, O'Connor R, Duronio V. Proliferation of pulmonary interstitial fibroblasts is mediated by transforming growth factor-beta1-induced release of extracellular fibroblast growth factor-2 and phosphorylation of p38 MAPK and JNK. J Biol Chem. 2005;280(52):43000–9. 10.1074/jbc.M510441200 16246848

[43] Liu T, Warburton RR, Guevara OE, Hill NS, Fanburg BL, Gaestel M, et al Lack of MK2 inhibits myofibroblast formation and exacerbates pulmonary fibrosis. Am J Respir Cell Mol Biol. 2007;37(5):507–17. 10.1165/rcmb.2007-0077OC 17600313 PMC2048679

[44] Kolosova I, Nethery D, Kern JA. Role of Smad2/3 and p38 MAP kinase in TGF-β1-induced epithelial-mesenchymal transition of pulmonary epithelial cells. J Cell Physiol. 2011;226(5):1248–54. 10.1002/jcp.22448 20945383 PMC3043117

[45] Murray LA, Chen Q, Kramer MS, Hesson DP, Argentieri RL, Peng X, et al TGF-beta driven lung fibrosis is macrophage dependent and blocked by Serum amyloid P. Int J Biochem Cell Biol. 2011;43(1):154–62. 10.1016/j.biocel.2010.10.013 21044893

[46] Li D, Guabiraba R, Besnard AG, Komai-Koma M, Jabir MS, Zhang L, et al IL-33 promotes ST2-dependent lung fibrosis by the induction of alternatively activated macrophages and innate lymphoid cells in mice. J Allergy Clin Immunol. 2014;134(6):1422–32 e11. 10.1016/j.jaci.2014.05.011 24985397 PMC4258609

[47] Redente EF, Keith RC, Janssen W, Henson PM, Ortiz LA, Downey GP, et al Tumor necrosis factor-alpha accelerates the resolution of established pulmonary fibrosis in mice by targeting profibrotic lung macrophages. Am J Respir Cell Mol Biol. 2014;50(4):825–37. 10.1165/rcmb.2013-0386OC 24325577 PMC4068926

[48] Wang Y, Kuai Q, Gao F, Wang Y, He M, Zhou H, et al Overexpression of TIM-3 in Macrophages Aggravates Pathogenesis of Pulmonary Fibrosis in Mice. Am J Respir Cell Mol Biol. 2019;61(6):727–36. 10.1165/rcmb.2019-0070OC 31162951

[49] Balli D, Zhang Y, Snyder J, Kalinichenko VV, Kalin TV. Endothelial cell-specific deletion of transcription factor FoxM1 increases urethane-induced lung carcinogenesis. Cancer Res. 2011;71(1):40–50. 10.1158/0008-5472.CAN-10-2004 21199796 PMC3075588

[50] Raghu G, Collard HR, Egan JJ, Martinez FJ, Behr J, Brown KK, et al An official ATS/ERS/JRS/ALAT statement: idiopathic pulmonary fibrosis: evidence-based guidelines for diagnosis and management. Am J Respir Crit Care Med. 2011;183(6):788–824. 10.1164/rccm.2009-040GL 21471066 PMC5450933

[51] Noble PW, Barkauskas CE, Jiang D. Pulmonary fibrosis: patterns and perpetrators. J Clin Invest. 2012;122(8):2756–62. 10.1172/JCI60323 22850886 PMC3408732

[52] Cai Y, Bolte C, Le T, Goda C, Xu Y, Kalin TV, et al FOXF1 maintains endothelial barrier function and prevents edema after lung injury. Sci Signal. 2016;9(424):ra40 10.1126/scisignal.aad1899 27095594

[53] Cheng X-H, Black M, Ustiyan V, Le T, Fulford L, Sridharan A, et al SPDEF Inhibits Prostate Carcinogenesis by Disrupting a Positive Feedback Loop in Regulation of the Foxm1 Oncogene. PLoS Genet. 2014;10(9):e1004656 10.1371/journal.pgen.1004656 25254494 PMC4177813

[54] Wang IC, Snyder J, Zhang Y, Lander J, Nakafuku Y, Lin J, et al Foxm1 mediates cross talk between Kras/mitogen-activated protein kinase and canonical Wnt pathways during development of respiratory epithelium. Mol Cell Biol. 2012;32(19):3838–50. 10.1128/MCB.00355-12 22826436 PMC3457538

[55] Xia H, Ren X, Bolte CS, Ustiyan V, Zhang Y, Shah TA, et al Foxm1 regulates resolution of hyperoxic lung injury in newborns. Am J Respir Cell Mol Biol. 2015;52(5):611–21. 10.1165/rcmb.2014-0091OC 25275225 PMC4491137

[56] Zaynagetdinov R, Sherrill TP, Kendall PL, Segal BH, Weller KP, Tighe RM, et al Identification of myeloid cell subsets in murine lungs using flow cytometry. Am J Respir Cell Mol Biol. 2013;49(2):180–9. 10.1165/rcmb.2012-0366MA 23492192 PMC3824033

[57] Westcott DJ, Delproposto JB, Geletka LM, Wang T, Singer K, Saltiel AR, et al MGL1 promotes adipose tissue inflammation and insulin resistance by regulating 7/4hi monocytes in obesity. J Exp Med. 2009;206(13):3143–56. 10.1084/jem.20091333 19995956 PMC2806469

[58] Suzuki T, Arumugam P, Sakagami T, Lachmann N, Chalk C, Sallese A, et al Pulmonary Macrophage Transplantation Therapy. Nature. 2014;514(7523):450–4. 10.1038/nature13807 25274301 PMC4236859

