# Supplementary figures and images for: Loss of FOXM1 in macrophages promotes pulmonary fibrosis by activating p38 MAPK signaling pathway
Source: PLoS Genet. 2020 Apr 9;16(4):e1008692. doi: 10.1371/journal.pgen.1008692 (PMC7173935; doi:10.1371/journal.pgen.1008692)

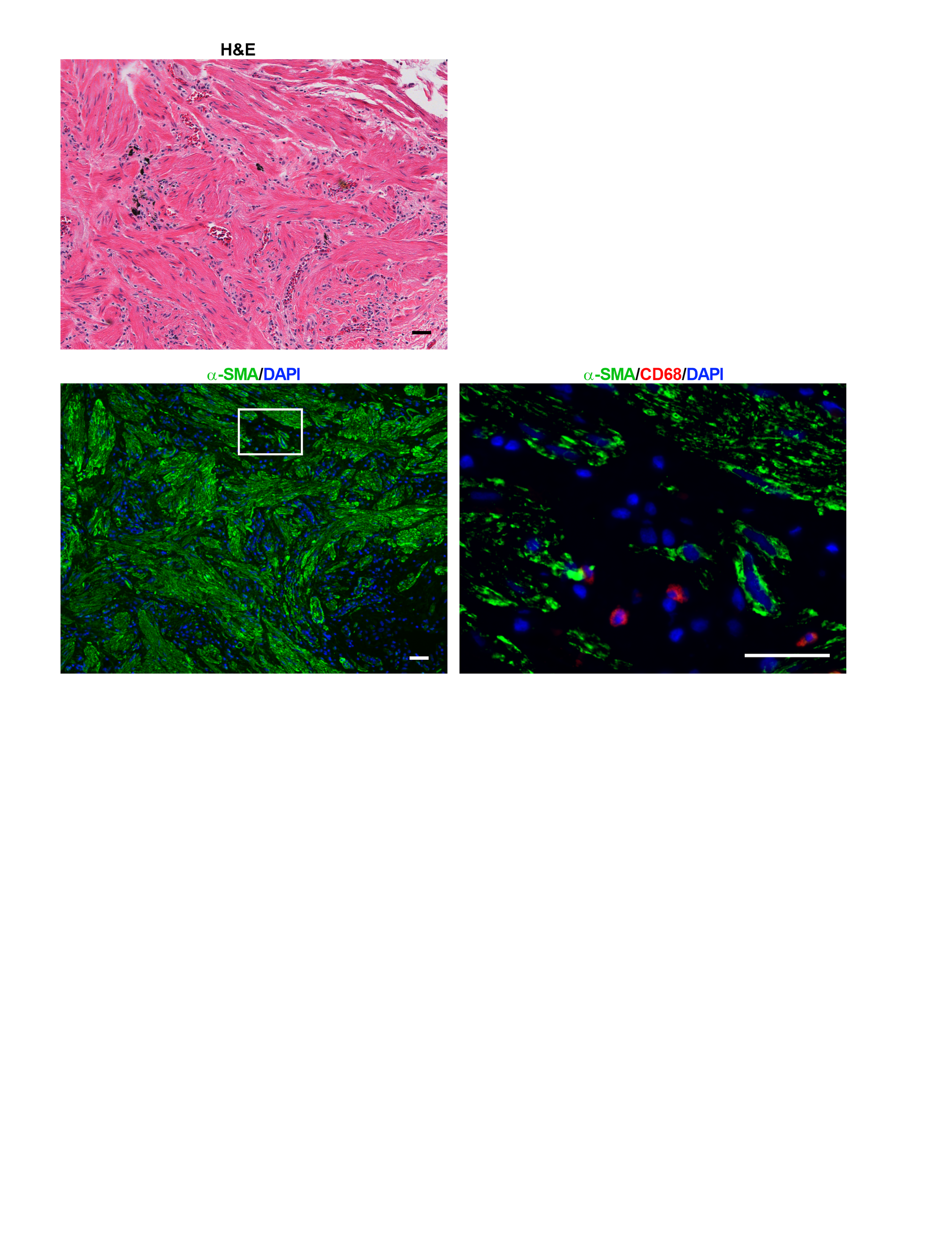

Supplement: S1 Fig — Fibrotic lesions in IPF patient lung sections were identified based on the characteristic histology using H&E staining (top panel). Immunostaining shows the presence of activated myofibroblasts positive for αSMA (green, bottom left panel) and the presence of CD68+ macrophages (red) in the fibrotic lesions of IPF lungs. (TIF) [file pgen.1008692.s001.tif]

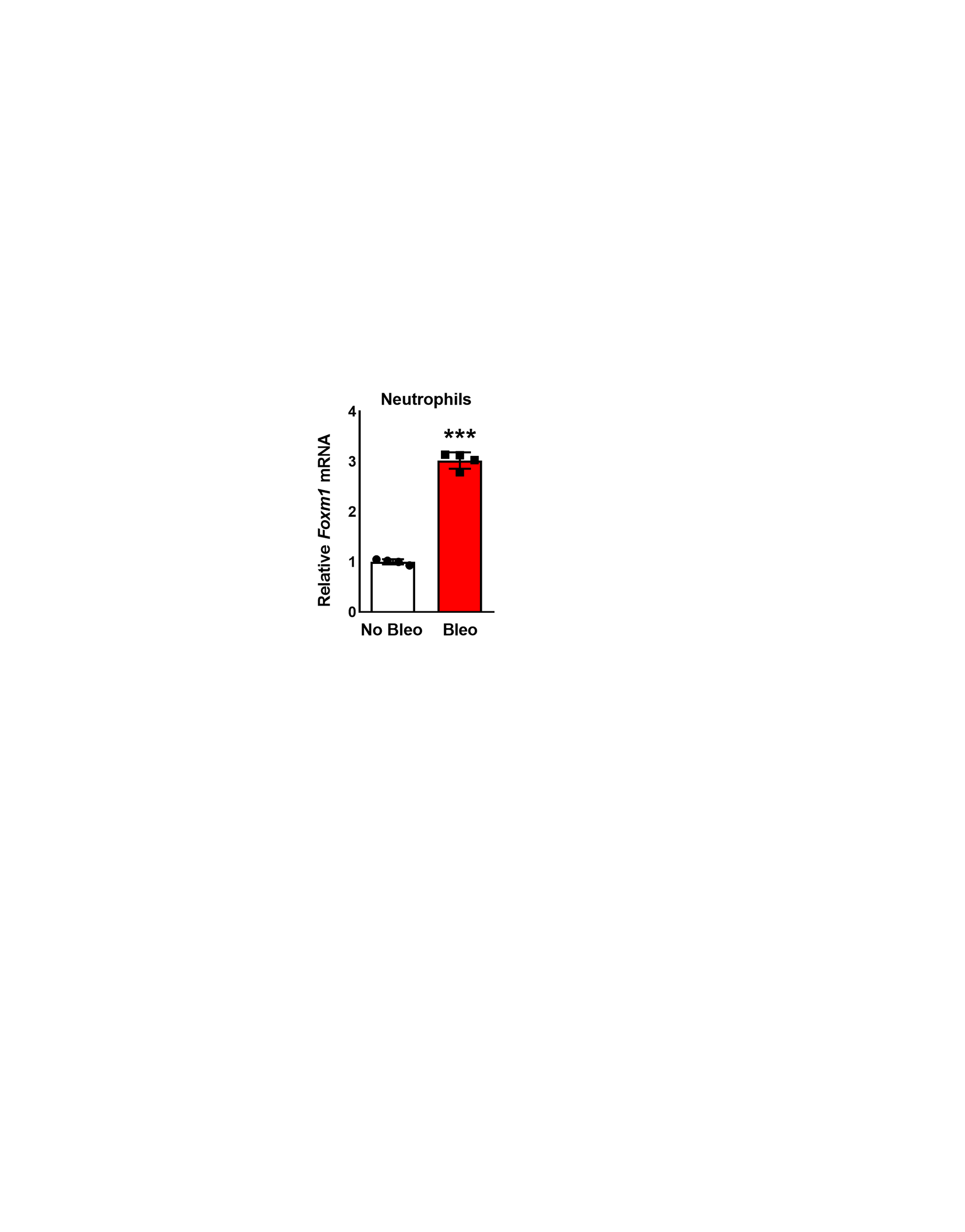

Supplement: S2 Fig — Neutrophils were isolated from bleomycin-treated or saline-treated WT lungs using Ly6G magnetic beads. qRT-PCR was used to measure Foxm1 mRNA. N = 8 mice per group. Actb mRNA was used for normalization. *** = P < 0.001, by Student’s t-test. (TIF) [file pgen.1008692.s002.tif]

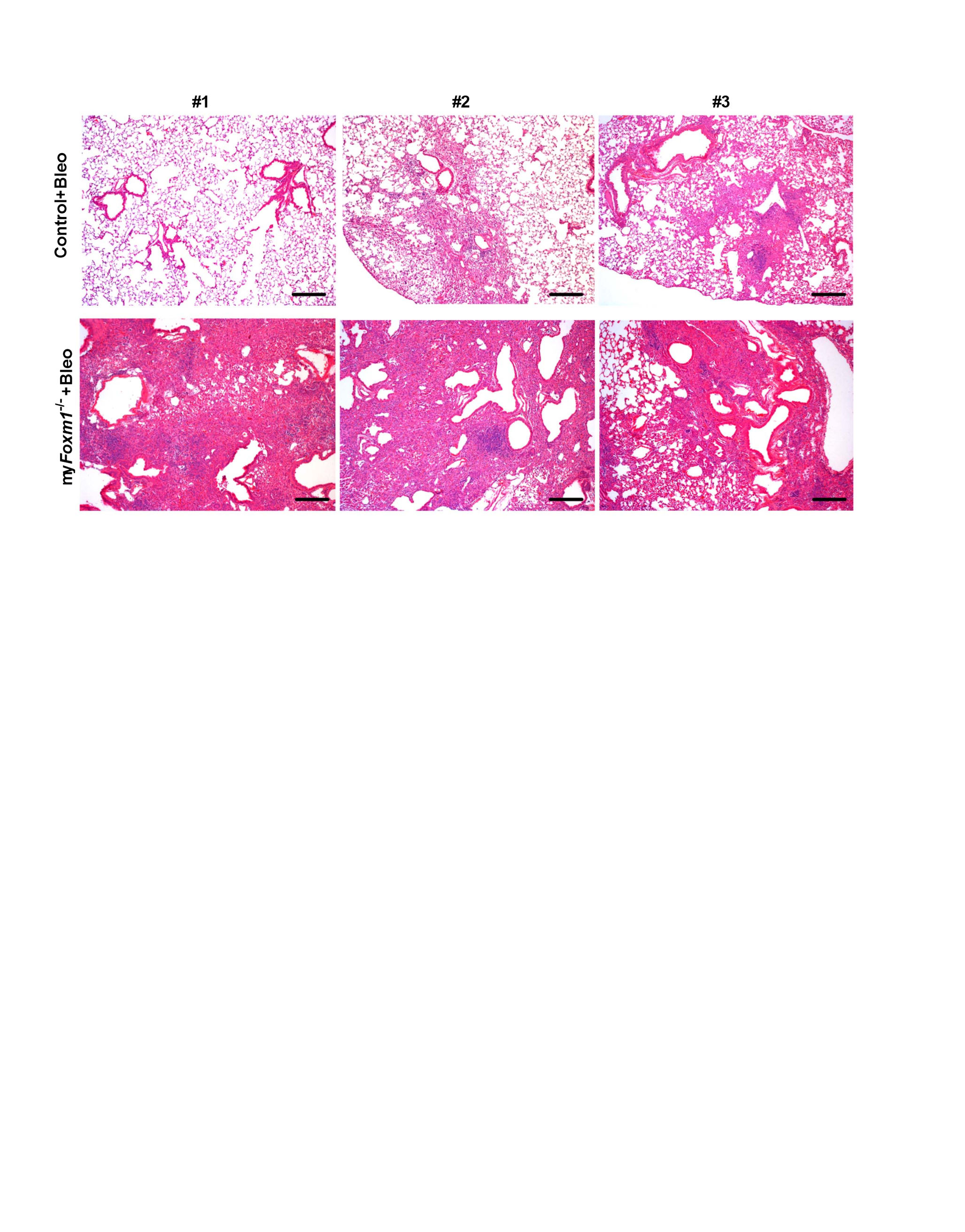

Supplement: S3 Fig — 8–10 weeks old myFoxm1-/- and Foxm1fl/fl mice were treated with bleomycin (1U/kg; once a week for three weeks). Mice were sacrificed, and lungs were collected one week after the final bleomycin treatment. H&E staining of lungs of 3 individual mice per group shows increased pulmonary fibrosis in bleomycin-treated myFoxm1-/- mice (bottom panels) compared to control bleomycin-treated mice (top panels). Scale bar = 200μm. (TIF) [file pgen.1008692.s003.tif]

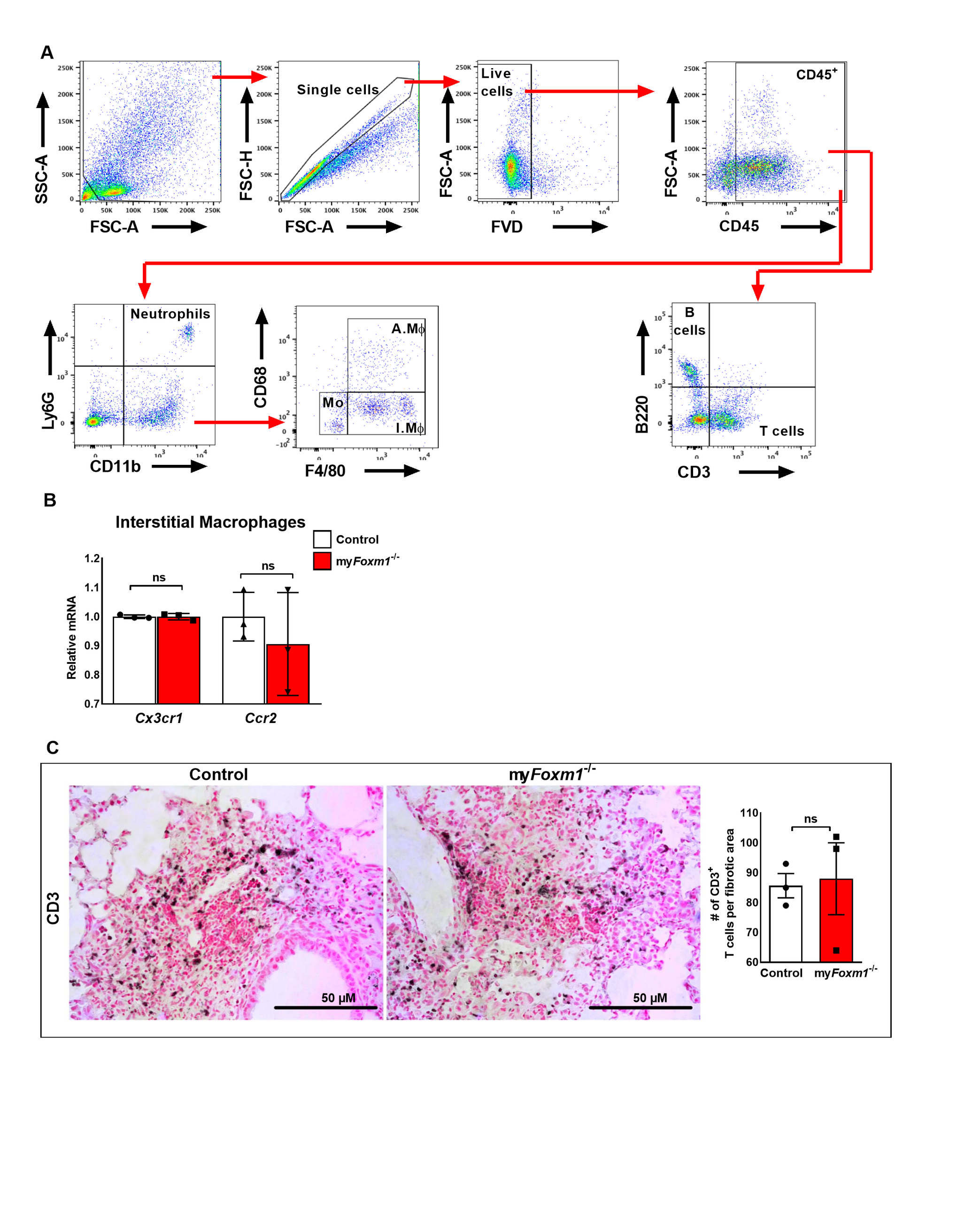

Supplement: S4 Fig — (A) Lung cell suspensions from bleomycin-treated mice were used for flow cytometry. Representative gating strategy for flow cytometry is shown. Single, live cells were gated to identify neutrophils (CD45+ CD11b+ Ly6G+), monocytes (CD45+ CD11b+ Ly6G- F4/80low), alveolar macrophages (CD45+ CD11b+ Ly6G- F4/80hi CD68hi), interstitial macrophages (CD45+ CD11b+ Ly6G- F4/80hi CD68low), B cells (CD45+ B220+), and T cells (CD45+ CD3+). (B) No difference in Cx3cr1 and Ccr2 mRNAs was found in interstitial macrophages FACS-sorted from bleomycin treated myFoxm1-/- mice compared to controls. qRT-PCR was used to measure mRNAs. Actb mRNA was used for normalization. N = 3 mice per group. (C) No difference was found in the number of T cells in bleomycin treated myFoxm1-/- lungs compared to controls. Number of CD3+ T cells were counted in 5 random fields and presented as mean ± SEM (n = 3 mice per group). Scale bar = 50μm. (TIF) [file pgen.1008692.s004.tif]

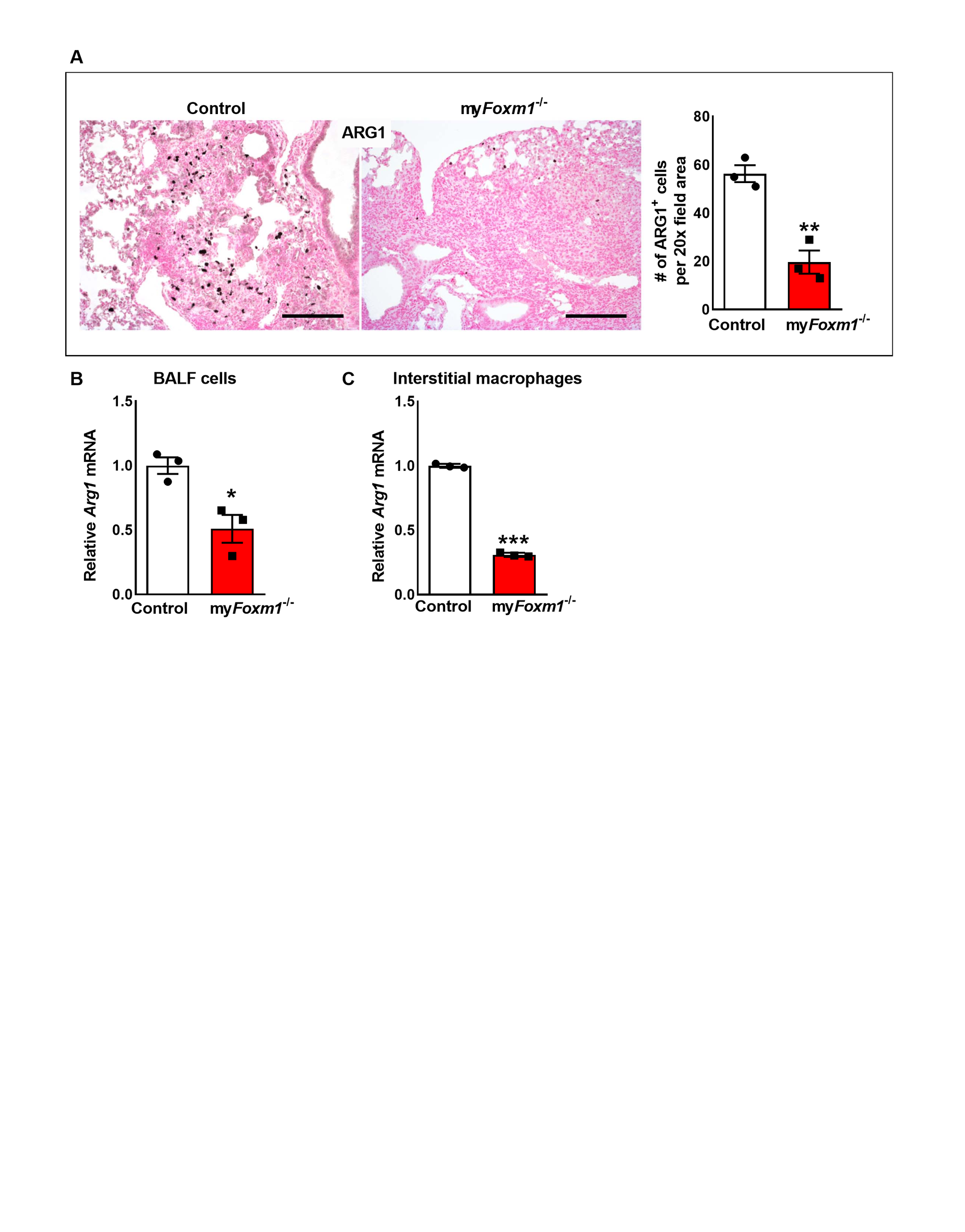

Supplement: S5 Fig — (A) Decreased expression of ARG1 is found in bleomycin-treated lungs of myFoxm1-/- mice compared to controls. Number of ARG1+ cells were counted in 5 random fields and presented as mean ± SEM (n = 3 mice per group). Scale bar = 200μm. (B) Arg1 mRNA is decreased in BALF cells isolated from bleomycin-treated myFoxm1-/- mice compared to controls as determined by qRT-PCR. Actb mRNA was used for normalization (n = 3 mice per group). (C) Arg1 mRNA is decreased in interstitial macrophages FACS-sorted from bleomycin-treated myFoxm1-/- mice compared to controls as determined by qRT-PCR. Actb mRNA was used for normalization (n = 3 mice per group). * = P > 0.05; ** = P > 0.01, *** = P < 0.001, by Student’s t-test. (TIF) [file pgen.1008692.s005.tif]

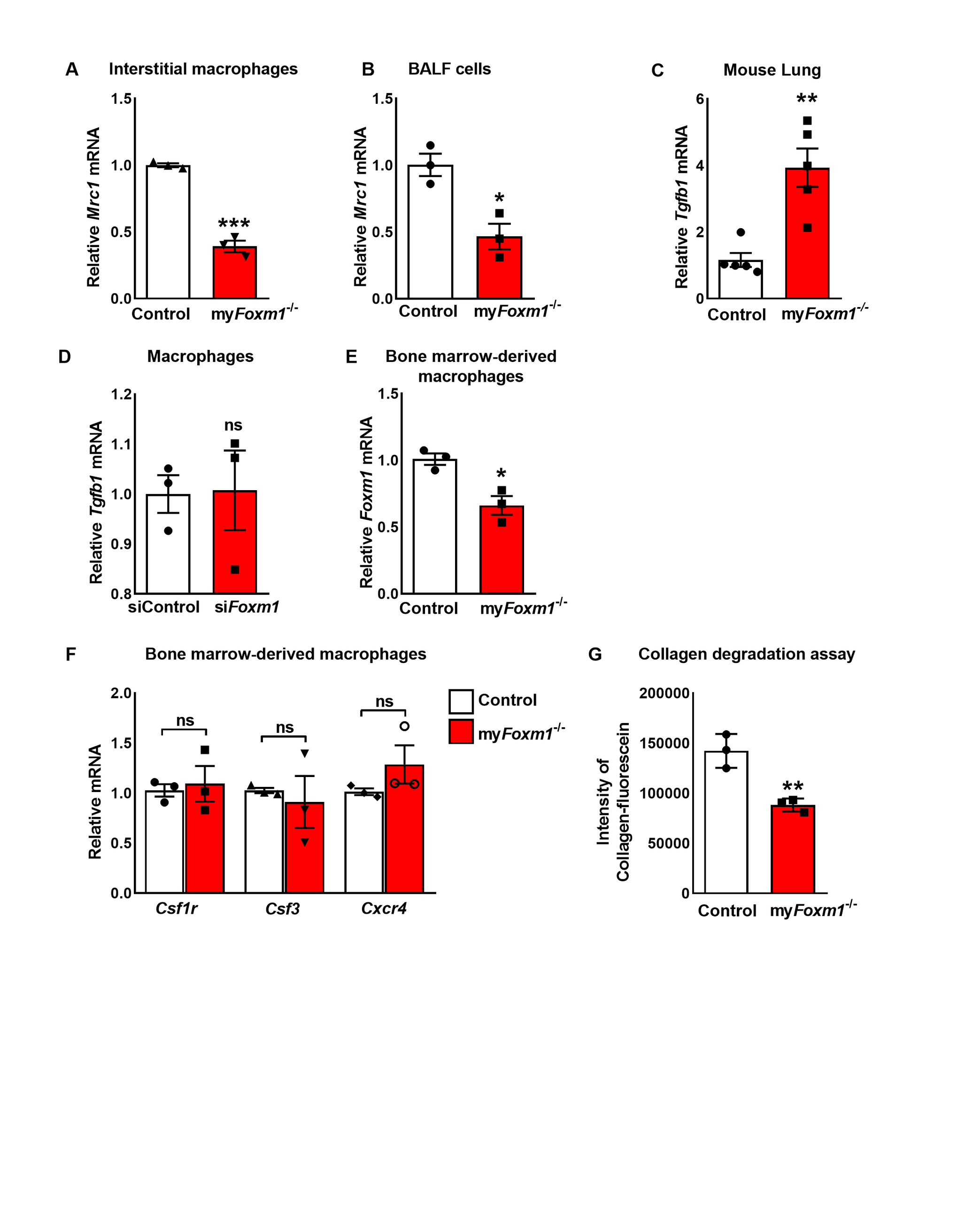

Supplement: S6 Fig — (A) Mrc1 mRNA is decreased in interstitial macrophages FACS-sorted from bleomycin-treated myFoxm1-/- mice compared to controls as determined by qRT-PCR. Actb mRNA was used for normalization. N = 3 mice per group. (B) Mrc1 mRNA is decreased in BALF cells isolated from bleomycin-treated myFoxm1-/- mice compared to controls as determined by qRT-PCR. Actb mRNA was used for normalization. N = 3 mice per group. (C) Tgfb1 mRNA is increased in total lung RNA from bleomycin-treated myFoxm1-/- mice as determined by qRT-PCR. Actb mRNA was used for normalization (n = 5 mice per group). (D) Depletion of Foxm1 in mouse macrophages did not change Tgfb1 mRNA as shown by qRT-PCR. RAW264.7 cells were transfected with control siRNA or siFoxm1 and mRNA levels were measured by qRT-PCR. Actb mRNA was used for normalization (n = 3). (E) Foxm1 mRNA is decreased in bone-marrow derived macrophages from myFoxm1-/- mice compared to controls as determined by qRT-PCR. Actb mRNA was used for normalization. N = 3 mice per group. (F) Csfr1, Csf3, and Cxcr4 mRNA levels are unchanged in bone-marrow derived macrophages from treated myFoxm1-/- mice as compared to control mice as determined by qRT-PCR. Actb mRNA was used for normalization (n = 3 mice per group). (G) Collagen degradation is decreased in bone-marrow derived macrophages from myFoxm1-/- mice compared to controls. The fluorescent signal from control wells containing only collagen was subtracted from that from wells containing macrophages and collagen. Assay was done in triplicates using bone-marrow derived macrophages from 3 individual mice per group. * = P > 0.05; ** = P > 0.01, *** = P < 0.001, by Student’s t-test. (TIF) [file pgen.1008692.s006.tif]

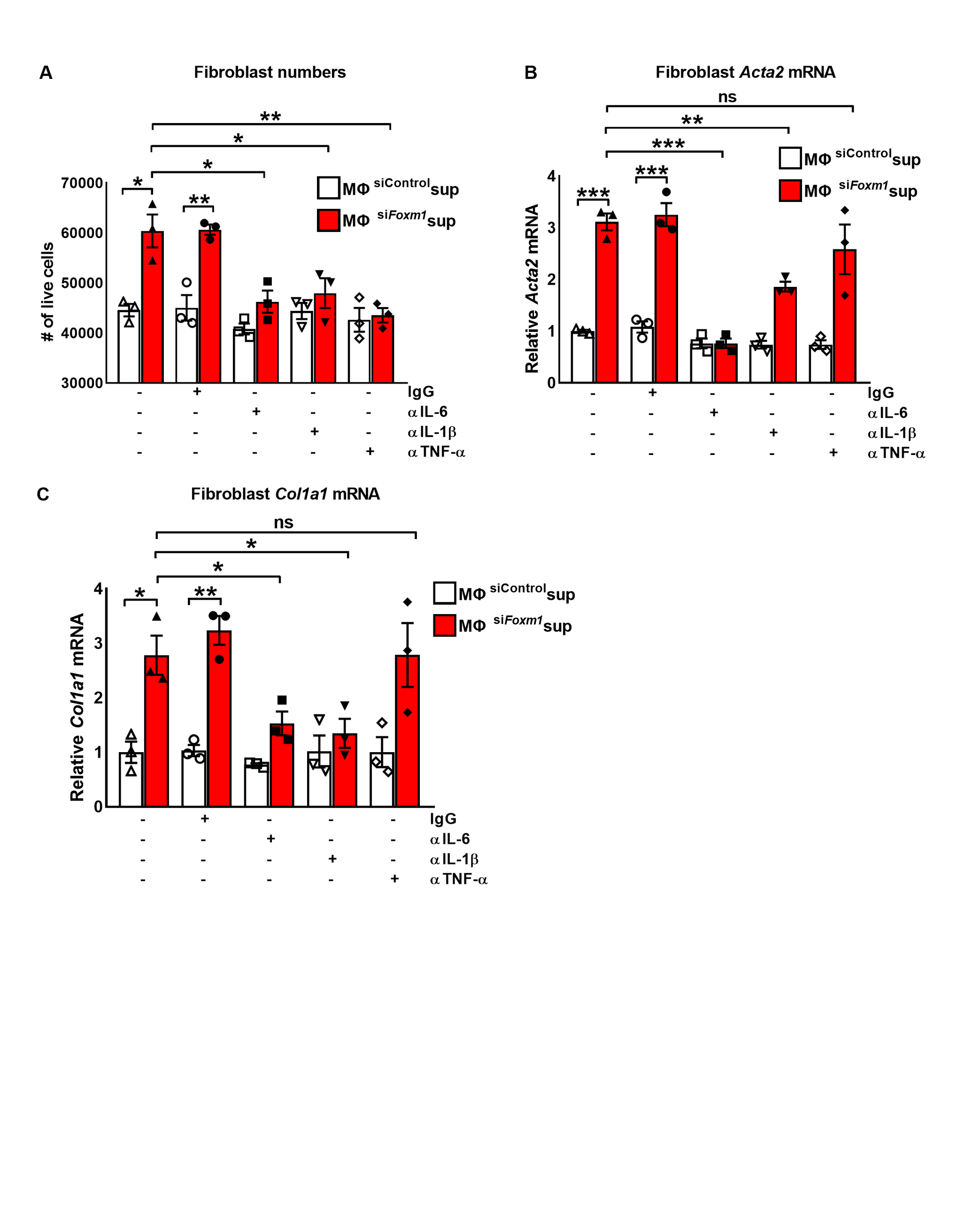

Supplement: S7 Fig — (A) Supernatant from Foxm1-deficient macrophages decreases fibroblast survival through IL-6, IL-1β, and TNF-α. The NIH-3T3 fibroblasts were cultured in supernatant obtained from RAW264.7 macrophages transfected with control siRNA or siFoxm1. IL-6, IL-1β, and TNF-α were inhibited in the cell culture using neutralizing Abs. 48 hours after addition of supernatant, the numbers of live fibroblasts were counted using trypan blue (n = 3). Neutralizing antibody against IgG was used as control. (B-C) Supernatant from Foxm1-deficient macrophages decreases Acta2 and Col1a1 mRNAs in fibroblasts through IL-6 and IL-1β as shown by qRT-PCR. mRNA levels in fibroblasts were analyzed by qRT-PCR. Actb mRNA was used for normalization (n = 3). (TIF) [file pgen.1008692.s007.tif]

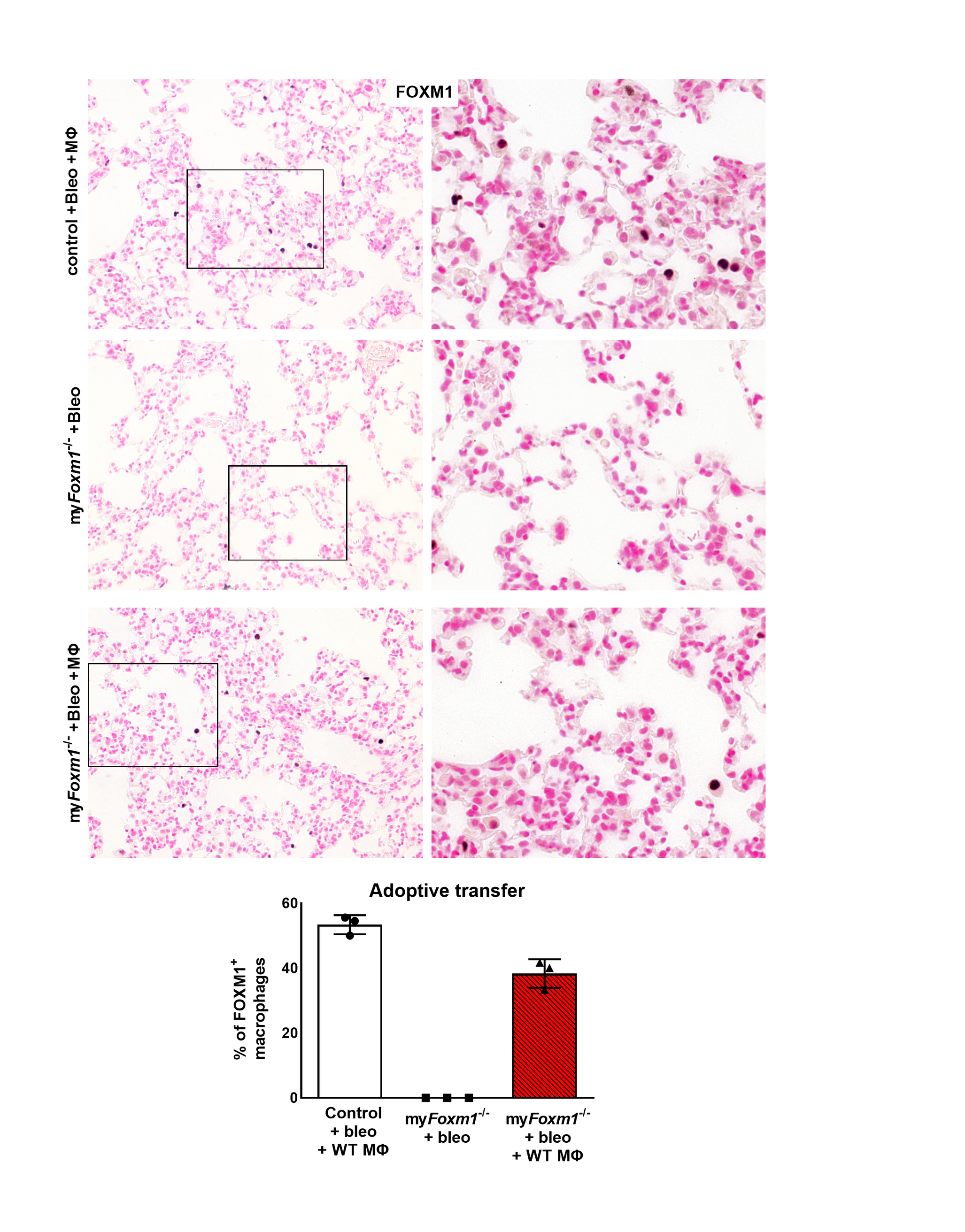

Supplement: S8 Fig — Immunostaining shows FOXM1-positive macrophages in bleomycin-treated control lungs (top panels) and myFoxm1-/- lungs (bottom panels) after adoptive transfer of WT monocytes. No FOXM1-positive macrophages are found in bleomycin-treated myFoxm1-/- lungs without adoptive transfer (middle panels). Control and myFoxm1-/- mice were treated twice weekly with bleomycin (1.0 U / Kg). One week after treatment, bone marrow-derived monocytes were injected (I.V.) into both groups of mice. Mice were sacrificed one week after the adoptive transfer. Numbers of FOXM1+ cells were counted in 5 random fields and presented as mean ± SEM. N = 3 mice per group. (TIF) [file pgen.1008692.s008.tif]
