# Supplementary material for: Loss of FOXM1 in macrophages promotes pulmonary fibrosis by activating p38 MAPK signaling pathway
Source: PLoS Genet. 2020 Apr 9;16(4):e1008692. doi: 10.1371/journal.pgen.1008692 (PMC7173935; doi:10.1371/journal.pgen.1008692)
Supplement: S1 Table — (DOCX) [file pgen.1008692.s009.docx]

**Supplemental Table 1. Taqman probes**

| **Gene** | **Assay ID** |
| --- | --- |
| *Actb* | Mm00607939_g1 |
| *Foxm1* | Mm01184444_g1 |
| *Dusp1* | Mm00457274_g1 |
| *Aurkb* | Mm01718146_g1 |
| *Ccnb1* | Mm03053893_gH |
| *Ccnd1* | Mm00432359_m1 |
| *Cdc25b* | Mm00499136_m1 |
| *Arg1* | Mm00475988_m1 |
| *Pdgfb* | Mm01298578_m1 |
| *Tgfb1* | Mm01178820_m1 |
| *Il1b* | Mm00434228_m1 |
| *Mmp9* | Mm00442991_m1 |
| *Mmp12* | Mm00500554_m1 |
| *Tnf* | Mm00443258_m1 |
| *Cx3cr1* | Mm02620111_g1 |
| *IL-6* | Mm01210733_m1 |
| *Ccr2* | Mm00438270_m1 |
| *Mapk14* | Mm01301009_m1 |
| *Csf3* | Mm00438334_m1 |
| *Mrc1* | Mm01329362_m1 |
| *Cxcr4* | Mm01292123_m1 |
