# Supplementary material for: Loss of FOXM1 in macrophages promotes pulmonary fibrosis by activating p38 MAPK signaling pathway
Source: PLoS Genet. 2020 Apr 9;16(4):e1008692. doi: 10.1371/journal.pgen.1008692 (PMC7173935; doi:10.1371/journal.pgen.1008692)
Supplement: S2 Table — (DOCX) [file pgen.1008692.s010.docx]

**Supplemental Table 2. Antibodies**

| **Antibody** | **Dilution** | **Company** | **Catalogue number** |
| --- | --- | --- | --- |
| FOXM1 (C-20) | 1:100 | Santa Cruz Biotechnology | 502 |
| CD68 | Pre-diluted, ready to use | Life technologies | 08-0125 |
| MAC-3 | 1:50 | BD Pharmigen | 550292 |
| α-SMA | 1:10,000 | Sigma | M5528 |
| Phospho-p38 MAPK | 1:50 | Cell Signaling Technologies | 4631 |
| Total p38 MAPK | 1:200 | Abcam | 31828 |
| DUSP1 | 1:100 | Abcam | 195261 |
| IL-1𝛽 | 1:100 | R&D | AF-401 |
